# Cost-effectiveness of acupuncture versus standard care for pelvic and low back pain in pregnancy: A randomized controlled trial

**DOI:** 10.1371/journal.pone.0214195

**Published:** 2019-04-22

**Authors:** Stephanie Nicolian, Thibault Butel, Laetitia Gambotti, Manon Durand, Antoine Filipovic-Pierucci, Alain Mallet, Mamadou Kone, Isabelle Durand-Zaleski, Marc Dommergues

**Affiliations:** 1 AP-HP, Service de Gynécologie-Obstétrique- Hôpital Universitaire Pitié-Salpêtrière- Charles Foix, Paris, France; 2 AP-HP, Centre intégré de médecine chinoise—Hôpital Universitaire Pitié-Salpêtrière- Charles Foix, Paris, France; 3 INSERM and AP-HP, CIC-1421, Hôpital Universitaire Pitié-Salpêtrière- Charles Foix, Paris, France; 4 AP-HP Unité de recherche clinique en économie de la santé—hôpital Hôtel-Dieu, Paris, France; 5 AP-HP Unité de recherche Clinique, Hôpital Universitaire Pitié-Salpêtrière- Charles Foix, Paris, France; 6 Sorbonne Université, Paris, France; Ottawa Hospital Research Institute, CANADA

## Abstract

**Objective:**

To assess the cost-effectiveness of acupuncture for pelvic girdle and low back pain (PGLBP) during pregnancy.

**Design:**

Pragmatic-open-label randomised controlled trial.

**Setting:**

Five maternity hospitals

**Population:**

Pregnant women with PGLBP

**Method:**

1:1 randomization to standard care or standard care plus acupuncture (5 sessions by an acupuncturist midwife).

**Main outcome measure:**

Efficacy: proportion of days with self-assessed pain by numerical rating scale (NRS) ≤ 4/10. Cost effectiveness (societal viewpoint, time horizon: pregnancy): incremental cost per days with NRS ≤ 4/10. Indirect non-healthcare costs included daily compensations for sick leave and productivity loss caused by absenteeism or presenteeism.

**Results:**

96 women were allocated to acupuncture and 103 to standard care (total 199). The proportion of days with NRS ≤ 4/10 was greater in the acupuncture group than in the standard care group (61% vs 48%, p = 0.007). The mean Oswestry disability score was lower in the acupuncture group than with standard care alone (33 versus 38, Δ = 5, 95% CI: 0.8 to 9, p = 0.02). Average total costs were higher in the control group (€2947) than in the acupuncture group (€2635, Δ = —€312, 95% CI: -966 to +325), resulting from the higher indirect costs of absenteeism and presenteeism. Acupuncture was a dominant strategy when both healthcare and non-healthcare costs were included. Costs for the health system (employer and out-of-pocket costs excluded) were slightly higher for acupuncture (€1512 versus €1452, Δ = €60, 95% CI: -272 to +470).

**Conclusion:**

Acupuncture was a dominant strategy when accounting for employer costs. A 100% probability of cost-effectiveness was obtained for a willingness to pay of €100 per days with pain NRS ≤ 4.

## Introduction

Pelvic girdle and low back pain (PGLBP) is common during pregnancy affecting 5 to 76% of pregnant women, depending on clinical definition[1–4]. PGLBP is characterized by pain between the posterior iliac crest and the gluteal fold, particularly in the vicinity of the sacroiliac joints (SIJ) or in the lumbar region above the sacrum[5,6]. PGLBP has a complex, multifactorial and incompletely understood pathophysiology. Strenuous work, previous low back or pelvic girdle pain and weight gain during pregnancy are risk factors[6,7]. Pain increases with standing position and physical activity. Diagnosis relies on analysing functional complaints and performing basic pain provocation tests[5,6,8]. Symptoms tend to disappear after delivery, but may continue up to 3 years in about 20% of women[9].

Because it results in poor functional and quality of life scores [2,10–12],PGLP represents an economic burden, especially regarding indirect non-healthcare costs related to time lost for work and leisure[2].

As for treatment, European guidelines recommend individualized exercises focusing on stabilizing exercises, information and reassurance, and a limited choice of pain medications, with acetaminophen as first choice[8]. Over 50%-75% of women with PGLBP receive little or no intervention from healthcare providers[3,5,13]. There is some evidence of low back pain alleviation during pregnancy by acupuncture[14–20], but offering acupuncture as part of routine obstetrical care would result in extra costs for the health system and patients. Therefore, our purpose was to assess the cost-effectiveness of providing hospital acupuncture care for PLBP in pregnancy.

## Methods

### Trial design and participants

The Trial of Acupuncture for Pelvic and low back Pain in Pregnancy (TAPP) was a pragmatic randomized open-label trial, conducted with pregnant women from five maternity units from the Paris area (France) between 2012 and 2014. We advertised the trial by posters in waiting rooms. When doctors or midwifes found an eligible patient, they sent a datasheet to the Centre d’Investigation Clinique in which acupuncture was performed, and an appointment was given for inclusion visit.

Women were randomized in parallel to receive standard care or standard care plus acupuncture with a 1:1 allocation ratio. Inclusion criteria were a singleton pregnancy, maternal age 18 or older, gestational age between 16 and 34 weeks, low back pain for at least two weeks, with pain greater than 4 on a 10-point numerical rating scale (NRS) and at least one positive provocation test. Pregnant women were not included if they had obstetrical complications such as preeclampsia or a small for gestational age foetus, if they had pelvic or low back pain before pregnancy, or if they did not have social insurance coverage. Patients were not involved in the study design or funding. However, a preliminary unpublished survey showed that a majority of pregnant women with low back pain would have liked to benefit from acupuncture and that half of them declared they would accept to participate in a trial.

### Intervention

The intervention consisted in 5 acupuncture sessions, performed by an acupuncturist midwife (SN) on top of standard treatment. There were 2 sessions in the first week, followed by three weekly sessions. Additional sessions could be done at the patient’s request. Acupuncture points’ selection was based on pain location and traditional Chinese medicine (TCM) diagnosis of “Qi kidney deficiency” versus “blood stagnation” (S1 Table).Baseline acupoints were 40V, *Weizhong* and AShi points. We added points were as a function of pain location (S2 Table)

Points were needled bilaterally. The patient lied on her left side, using cushions to make her comfortable. We used Dong Bang needles (Acushop, Dongbang Needles, Chungnam, Korea) 25 or 40 mm in length and 0.25 mm in diameter. While inserting the needle, the operator performed manual stimulation to elicit the *Deqi* response. Needles were retained for 30 minutes per treatment.

Standard treatment in both groups consisted of pregnancy belt, lifestyle recommendations, and exercises, explained by the midwife in charge of the trial. Painkillers, rest and sick leaves were prescribed by the referring physician or midwife.

### Outcome

The primary endpoint was the percentage of days, between inclusion and delivery, with daily greatest pain self-assessed by numerical rating scale (NRS) ≤ 4/10

Secondary endpoints were:

- Last week worst pain self-assessed by numerical rating scale, at the week-5 visit, expressed as differences between groups and within each group by the difference between baseline and week-5,
- Last week worst pain measured at the week-5 visit by analogic rating scale expressed as differences between groups and within each group by the difference between baseline and week-5,
- Mean Oswestry disability index collected weekly between inclusion and delivery, measured by the Oswestry disability self-questionnaire,
- Percentage of days with a reduction of 2 points of the self-assessed NRS pain compared to baseline,
- Percentage of weeks with Oswestry disability index of 20/100 or lower,
- Percentage of weeks with a reduction of at least 15 points in the Oswestry index compared to baseline.

### Randomization

Women were randomized between acupuncture or control group with a central web based randomization system, using block randomization with a block size of four, an allocation ratio of 1:1 and stratification for hospital. The web-based randomization system generated a unique number with allocation code.

### Follow-up and data collection

Trial endpoints and healthcare utilization details were collected prospectively from the case report form filled by the investigator and from the patient’s logbook (self-assessment). At inclusion and five weeks later (week-5 visit), the investigator recorded pain location, and pain intensity by visual analogic rating scale (VAS). Patients filled self-questionnaires to assess pain intensity by numerical analogic rating scale (NRS) and disability (Oswestry disability self-questionnaire—OSW)[21]. The investigator was blinded to the results of the self-questionnaires.

At inclusion, the investigator gave patients a logbook on which they reported daily health outcomes and use of resources until delivery. They rated their greatest pain perceived using a ten point NRS. A day with a successful outcome was defined as a day with NRS of 4/10 or lower. Patients filled the Oswestry disability questionnaire weekly. Each week a research technician called patients in order to remind them to fill the logbook. After each session and at the week-5 visit the acupuncturist asked patients about bruising, fatigue, dizziness, headache using a standardized questionnaire. We assessed non-specific adverse events, retrospectively, by analysing the medical records after delivery. We based the estimation of costs on the logbook on which patients recorded the use of pain medications, hospitalisations, healthcare consultations and any analgesic practice. After delivery, we checked the duration of hospital stays in the medical records.

The prospective economic evaluation was concurrent with the randomized trial, in accordance with the CHEERS statement for single trial based studies[22]. All economic analyses were carried out on an intention to treat basis. The costs considered in the present analysis were collected from a societal viewpoint, according to the recommendations of the, the French National Health Authority (HAS). The time horizon was until the delivery. Direct medical costs included all outpatient or inpatient care from inclusion to delivery.

### Estimation of unit costs

Hospitalizations were valued using the hospital perspective, based on mean hospital costs per day, estimated by the French Agence Technique de l'Information sur l'Hospitalisation[23]. The cost of an acupuncture session in the protocol was valued using the average hourly wage of a midwife, €30. Painkillers, outpatient clinics, physiotherapy, ultrasound, medical transportation, acupuncture sessions outside the protocol were valued using health insurance reimbursement (https://www.ameli.fr). Out-of-pocket healthcare expenses were pregnancy belt, osteopathy, relaxation, yoga, gym, massages.

Indirect non-healthcare costs included daily compensations for sick leave and additional maternity leave paid by the health insurance [24]. We estimated daily compensations using adjusted average daily wage rates from the national statistics office[25] earnings survey and the thresholds for compensation referenced by the healthcare insurance. Also included were indirect costs of loss of production owing to absenteeism from work and presenteeism at work for patients with a paid job. Productivity loss related to presenteeism, i.e. attending work despite low back pain, was defined as time lost because of low back pain and was valued using adjusted average hourly wage rates. Loss of working time due to presenteeism was estimated for all patients to one hour per day with pain NRS > 4 based on literature review[26,27]. Details of individual costs for the different payers are available as supplementary material (S3 Table).

### Cost-effectiveness analysis

A cost-effectiveness analysis was conducted to estimate incremental costs per incremental day with pain NRS ≤ 4, per difference between baseline and week-5 (W5) NRS, or per incremental week with OSW ≤ 20. Incremental costs were the difference in mean per-patient costs between groups. We assessed the cost-effectiveness of acupuncture treatment versus standard treatment alone until delivery, using imputation for missing data. Costs were not discounted due to the short time horizon (between inclusion to delivery). Incremental cost-effectiveness ratios (ICER) were calculated, defined as the ratio between net total costs and net effects expressed in days with pain NRS ≤ 4: (Cost acupuncture− Cost control) / (Effectiveness acupuncture − Effectiveness control). We performed probabilistic sensitivity analyses and represented graphically the bootstrapped cost-effect pairs on a cost effectiveness plane. Acceptability curves were calculated, which showed the probability that a treatment is cost effective at a specific ceiling ratio [28–30].

### Missing values and imputation methods

Rare missing observations for baseline variables were imputed using chained equations. We used a two-step chained equations method to impute rare missing data on healthcare resources use and work stoppage. The Sensitivity analysis was performed without imputation.

For more frequent missing data (more than ten percent), i.e. for pain NRS and Oswestry score we suspected data to be not missing at random (NMAR), thus resulting in incidental sample selection. For these, we implemented a Heckman two-step procedure for the imputation of missing data, in order to correct for the suspected selection bias[31–34]. This two-step equation was run by using a mixed model for longitudinal data with both random slope and parameter.

### Statistical methods

#### Sample size calculation

The calculation of the number of patients to enrol in the study required a numerical estimation of the primary endpoint. Based on data from Elden ^16^, we assumed, that four weeks after enrolment, women in the control group would present an average maximum pain of 5/10, and women in the acupuncture group an average maximum pain of 3/10. Using the inter quartiles of reported by Elden et al, we estimated the standard deviation of the pain at baseline and at 4 weeks would be 0.21. We further assumed that women in the control group would exhibit a constant mean pain level of 0.5 standard deviation (SD 0.21) while women in the acupuncture group would show their mean pain level drop to 0.3 (SD 0.21) at 4 weeks after inclusion and raise to 0.4 at the end of follow-up. An additive random effect, based on a random walk with a 0.003 per day standard deviation was considered to account for intra individual variability. Based on these figures and on the ability to detect a clinically relevant difference of 25% in percentage of days with pain NRS ≤ 4 between groups, we estimated that 150 patients in each group would give a power of 80% and a two sided α-risk of 5%.

#### Data analysis

Categorical data were reported as frequencies, and continuous data are reported as mean ± standard deviation (SD). Comparisons of clinical endpoints between groups were standardized on a theoretical follow-up duration of 80 days. Discrete variables were compared using the Fisher exact test. Normally distributed continuous variables were compared using Student t test, and non-normally distributed data were compared using the Wilcoxon rank-sum test. Cost data were reported as mean values and were compared using a t-test. Bootstrapping (2000 replicates) was used to estimate uncertainty in the joint distribution of costs, ENS and OSW for each treatment group; all the 95% confidence intervals (95% CI) were estimated with this bootstrap technique.

All analyses were performed with R version 3.3.1 *(The R Core Team*. *R*: *2016)*.

### Ethics approval

The study was approved by the ethic review board “Comité de Protection des Personnes Ile de France VI”. Decision number: IDRCB: 2012-A00240-43. Date of decision: July 2nd 2012.

All patients provided written informed consent, after receiving verbal and written information specifying the design of the study, the duration of their participation, practical issues on the trial, and the fact they could withdraw their consent at any time.

### Registration

The protocol was registered on clinicaltrials.gov under reference NCT01848587. We registered on ClinicalTrials.gov on March 6th 2013, whereas the trial commenced in September 2012. This delay was due to an error in managing the trial. We made no change in the protocol between inclusion of the first participant and registration on ClinicalTrials.gov. We confirm that all ongoing and related trials for this intervention are registered.

## Results

**Fig 1:**
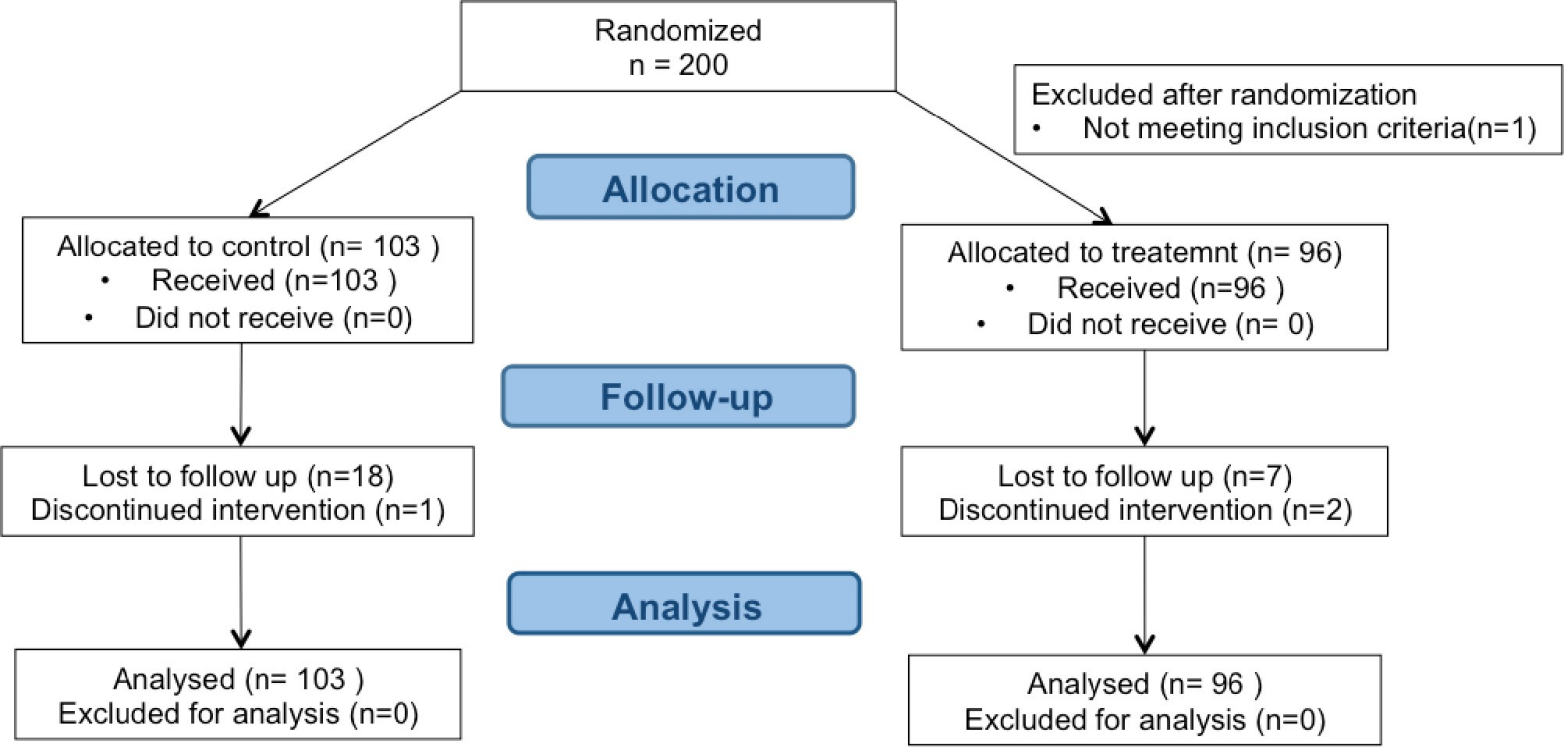
Flow chart.

During the study period, there were approximately 14800 deliveries per year in the maternity hospitals that recruited patients for the trial. Two hundred patients were randomized between October 2012 and September 2014: 96 patients to acupuncture group and 104 to standard treatment (control group). One patient was excluded after randomization because not fulfilling the inclusion criteria. Overall, 174 patients (87%) were followed up until delivery (Fig 1). All data of patients who withdrew from the trial were included in the analysis until the time of withdrawal, after which we used the imputation methods described above for missing data. We recorded no or rare missing data (< 5%) for baseline variables. Complete cost data were available for 86 (90%) patients in the acupuncture group and 81 (79%) in the standard care group. Missing data of the for daily pain record and weekly Oswestry score were more frequent in the control group than in the acupuncture group, respectively 31% vs 21% (pain NRS) and 37% vs 26% (Oswestry score). In the acupuncture group, 20 women requested extra sessions, with a total of 30 extra-sessions. Forty-seven women missed at least one session. The average time between inclusion and delivery was 81.1 days in acupuncture group and 84.7 days in control group. The mean follow-up was respectively 75.4 and 72.0 days.

Baseline clinical data are displayed on Table 1.

**Table 1:** Baseline clinical data.

		Acupuncture (n = 96)	Control (n = 103)
Age in years, mean (SD)		31 (5.2)	30.7 (4.6)
Gestational age at inclusion, in weeks, mean (SD)		28 (4.7)	27.4 (4.2)
Pre gestational BMI (kg/m ^2^) mean (SD)		23.7 (4.4)	24.1 (5.3)
Parity	*Para 0*	47 (49%)	45 (44%)
*Para 1*		32 (33%)	37 (36%)
*Para ≥2*		17 (18%)	21 (20%)
Scarred uterus		10 (10.4%)	8 (7.8%)
Hospital	*Pitié-Salpêtrière*	60 (62.5%)	62 (60.2%)
	*Kremlin Bicêtre*	19 (19.8%)	21 (20.4%)
	*Trousseau*	7 (7.3%)	7 (6.8%)
	*Tenon*	5 (5.2%)	4 (3.9%)
	*Necke*	4 (4.2%	4 (3.9%)
	*Other*	1 (1%)	5 (4.9%)
Professional in charge *Midwife*		69 (71.9%)	90 (87.4%)
*Doctor*		25 (26%)	12 (11.7%)
*Both*		2 (2.1%)	1 (1%)
Gestational age when pain started (weeks)		19.5 (5.6)	18.8 (5.1)
Pain location	*Low back pain (L3L5*	55 (57%)	58 (56%)
	*Back pain higher than L3*	23 (24%)	30 (29%)
	*Sacro-ileal pain*	69 (72%)	75 (73%)
	*Anterior pelvic pain*	35 (36%)	45 (44%)
	*Sciatica*	43 (45%)	36 (35%)
VAS pain	*Previous week worse pain*	72 (14)	72 (15)
at nclusion	*Previous week average pain*	54 (15)	54 (17)
mean(SD)	*Pain on the day of inclusion*	40 (26)	41 (26)
NRS pain	*Previous week worse pa*	7.4 (1.3)	7.4 (1.3)
at nclusion	*Previous week average pain*	5.6 (1.4)	5.5 (1.6)
mean(SD)	*Pain on the day of inclusion*	4.2 (2.5)	4.3 (2.6)
Mean Oswestry	Index at inclusion (SD)	36 (13)	38 (14)
Sick leave(N%)	at inclusion	44 (46.8%)	45 (45.5%)

VAS: visual analogic scale

NRS: numerical rating scale

Baseline clinical data were similar in the acupuncture group and in the control group

### Effectiveness of acupuncture

Acupuncture plus standard care was more effective than standard care alone regarding pain and disability (Table 2). Before imputation, the mean percentage of days with pain NRS ≤ 4 was 52% in the acupuncture group and 30% in the standard care group (p< 0.001). The mean number of days per patient with NRS ≤ 4 was 43 (95% CI: 36; 50) in the acupuncture group and 26 (95% CI: 20; 33) in controls. After imputation, the mean percentage of days with pain NRS ≤ 4 was 61% in the acupuncture group 48% in the standard care group (p = 0.007). The difference between groups was 13% (95% CI: 3.6; 22.1). The mean number of days per patient with NRS ≤ 4 was 51 (95% CI: 44; 58) in the acupuncture group and 42 (95% CI: 35; 50) in controls (estimation for a theoretical time of follow-up of 80 days for both groups: 38.3–95% CI: 43; 53—vs 48.6–95% CI: 33; 44).

Differences in pain intensity were clinically and statistically significant between the two groups. The pain numerical analogic rating scale at week 5 was lower in the acupuncture group than in the standard care group, resulting in an average reduction from baseline of 2.3 points in the acupuncture group versus 1.4 in the standard care group (Δ = 0.9, 95% CI: 0.2; 1.5, p = 0.008).

**Table 2:** Results for pain and disability.

	*Acupuncture (95% CI)*	Controls (95% CI)	Difference (95% CI)	p
Mean pain Numerical Rating Scale (NRS)* baseline	*7*,*4 (7*.*1; 7*.*6)*	7,5 (7.2; 7.7)	-	-
Percentage of days **with NRS ≤4/10, before imputation	*52% (49; 52)*	30% (24; 37)	22% (12; 31)	<0.001
Percentage of days with NRS ≤4/10, after imputation	*61% (54; 67)*	48% (41; 55)	13% (3.6; 22.1)	0.007
Mean NRS at week 5 before imputation	*5*.*1 (4*.*6; 5*.*7)*	6,6 (6.0; 7.0)	-	-
Difference in NRS between baseline and week 5 before imputation	*-2*.*2 (-2*.*7; -1*.*7)*	-0,9 (-1.5; -0.4)	1,2 (0.5; 2.0)	<0.001
Mean NRS at week 5, after imputation	*5 (4*.*6; 5*.*5)*	6 (5.5; 6.5)	-	-
Difference in NRS between baseline and week after imputation	*-2*.*3 (-2*.*8; -1*.*9)*	-1,4 (-1.9; -1.0)	0.9 (0.2; 1.5)	0.008
Difference in pain visual analogic scale (VAS) between baseline and week 5 after imputation	*-25(-30; -20)*	-17 (-22; -12)	8 (0.6; 15)	0.02
Mean Oswestry disability index (ODI), baseline	*36*.*0 (33*.*4; 38*.*7)*	38.2 (35.6; 41.0)	-	-
ODI at week 5 (SD),before imputation	*30*.*1 (26*.*0; 34*.*3)*	37.0(32.9; 41.1)	-	-
Mean difference in ODI between baseline and week 5 before imputation	*5*.*2 (2*.*2; 8*.*1)*	-0.3 (-3.8; 3.2)	5.5 (0.4; 9.7)	0.02
ODI at week 5 after imputation	*30*.*0 (26*.*4; 33*.*5)*	35.7(32.4; 38.9)	-	-
Mean difference in ODI between baseline and week 5 after imputation	*6*.*1 (3*.*5; 8*.*7)*	2.7 (0.0; 5.4)	3.5 (0.4; 9.7)	0.07
Mean ODI throughout pregnancy*, observed	*33 (29*.*5; 36*.*8)*	38.7 (35.6; 42.5)	-5.7(-11; -1)	0.02
Mean ODI throughout pregnancy*, after imputation	*33 (30*.*2; 36*.*6)*	38 (36.0; 41.2)	5 (0.8; 9)	0.02
Percentage* of weeks with ODI ≤20/100, before imputation	*21% (15; 27)*	10% (6; 15)	12% (3; 21)	0.003
Percentage*** of weeks with ODI ≤20/100, after imputation	*30% (25; 38)*	15% (11; 21)	7% (-2; 16)	<0.001

Regarding pain and disability, primary and secondary outcome measurements favoured acupuncture plus standard care (N = 96) vs. standard care alone (N = 103)

*NRS: Pain numerical rating scale, self-reported daily (worst pain in the past 24 hours).

**Percentage of days from inclusion to delivery. Results are given as raw observed data, and after imputation of missing data.

*** calculated between inclusion and delivery

As for disability, the differences in mean Oswestry disability scores between inclusion and delivery were also significantly in favour of acupuncture (Table 2)

We found no significant difference between groups for obstetrical and neonatal morbidity. In the acupuncture group, caesarean rate was 23%, mean birth weight was 3406g; four babies were admitted to neonatal care unit, 2 were born before 37 weeks. In the standard care group, caesarean rate was 20%, mean birth weight was 3327g, 7 babies were admitted to neonatal care unit, and one was born before 37 weeks (S5 Table).

### Adverse effects

Acupuncture-specific side effects occurred in 32 (33%) patients and included bruising (n = 24, 25%), fatigue (n = 9, 8%), dizziness (n = 1, 1%) and headache n = 1 (1%).

There was no difference between the acupuncture and the control group regarding non-specific adverse events (see additional table S4). The number of patients with at least one nonspecific adverse event was 29 (30%) in the acupuncture group and 30 (29%) in controls. In the acupuncture group, 10 patients were hospitalised because of a nonspecific adverse event, versus 9 in the control group. The total number of adverse events was 40 in the acupuncture group and 36 in controls. These events included cholestasis, gestational diabetes, hypertension/ preeclampsia, unexplained transient fever, urinary infection, viral infection, other infection, threatened premature labour, premature delivery (34–36 weeks), intrauterine growth restriction, and thrombocytopenia.

### Cost effectiveness analysis

From the societal perspective, average total costs were higher in the control group (€2947) than in the acupuncture group (€2635), with a difference of 312 euros (95% CI: €-966; €325). The lower cost in the acupuncture group was essentially due to indirect costs of absenteeism and presentism (S6 Table). With better effectiveness and lower cost the acupuncture strategy was dominant. When restricting the analysis to the viewpoint of the hospital, the ICER was €22 per additional day with pain NRS below 4 (Table 3).

**Table 3:** Total cost, total effectiveness, incremental cost, incremental effectiveness and incremental cost-effectiveness ratio. All costs are in 2016 €.

Variable expressed as mean or % (95%CI)	acupuncture	control	difference	Incremental cost effectiveness
Effectiveness (% of days with pain rating ≤4/10)	*61%*(54; 67)	48%(41; 55)	13%(3.6; 22.1)	
Total healthcare costs	1512(1286;1899)	1452(1247;1690)	60(39;199)	22 per additional day (dominant)
Total healthcare and nonhealthcare costs	2635(2269;3125)	2947(2494;3482)	312(-966; 325)	

**Fig 2:**
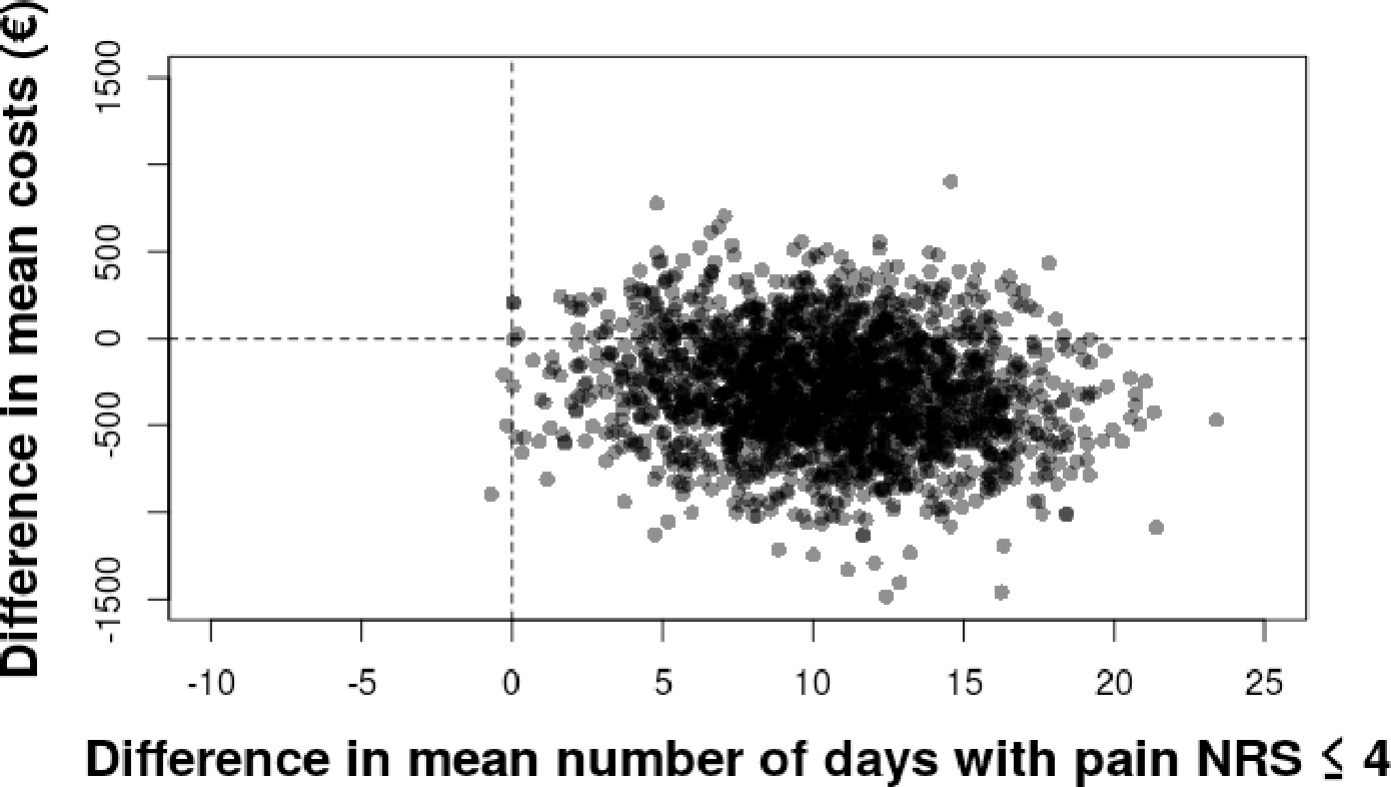
Cost effectiveness plane, outcome: number of days with pain numerical rating scale ≤ 4/10, costs from the societal perspective. We performed 2,000 bootstrap replications of the cost effectiveness ratio. The outcome was the number of days with pain NRS ≤ 4/10 between inclusion and delivery, expressed as a difference between acupuncture and standard care. All costs were taken into account. Acupuncture was always more effective and had a 70% probability of being less costly than routine care. Results for reduction on pain scales and weeks with disability were similar (S3 Fig). Fig 3 shows the probability of the intervention being cost effective using the base case data for a range of cost effectiveness ceilings. There was a nearly 100% probability that the cost per day of pain averted was below €100.

Fig 2 shows the cost effectiveness plane using as outcome the number of days with pain NRS ≤ 4/10 when comparing acupuncture and standard treatment groups. We performed 2,000 bootstrap replications of the cost effectiveness ratio for pain intensity. This showed that acupuncture in addition to usual care was always more effective and had a 70% probability of being less costly than routine care. We obtained similar results when the outcome was a decrease between baseline NRS and NRS at week 5 after inclusion (S1 Fig). When we took into account the hospital costs only however, acupuncture was more effective, but costlier that standard treatment alone (S2 Fig)

**Fig 3:**
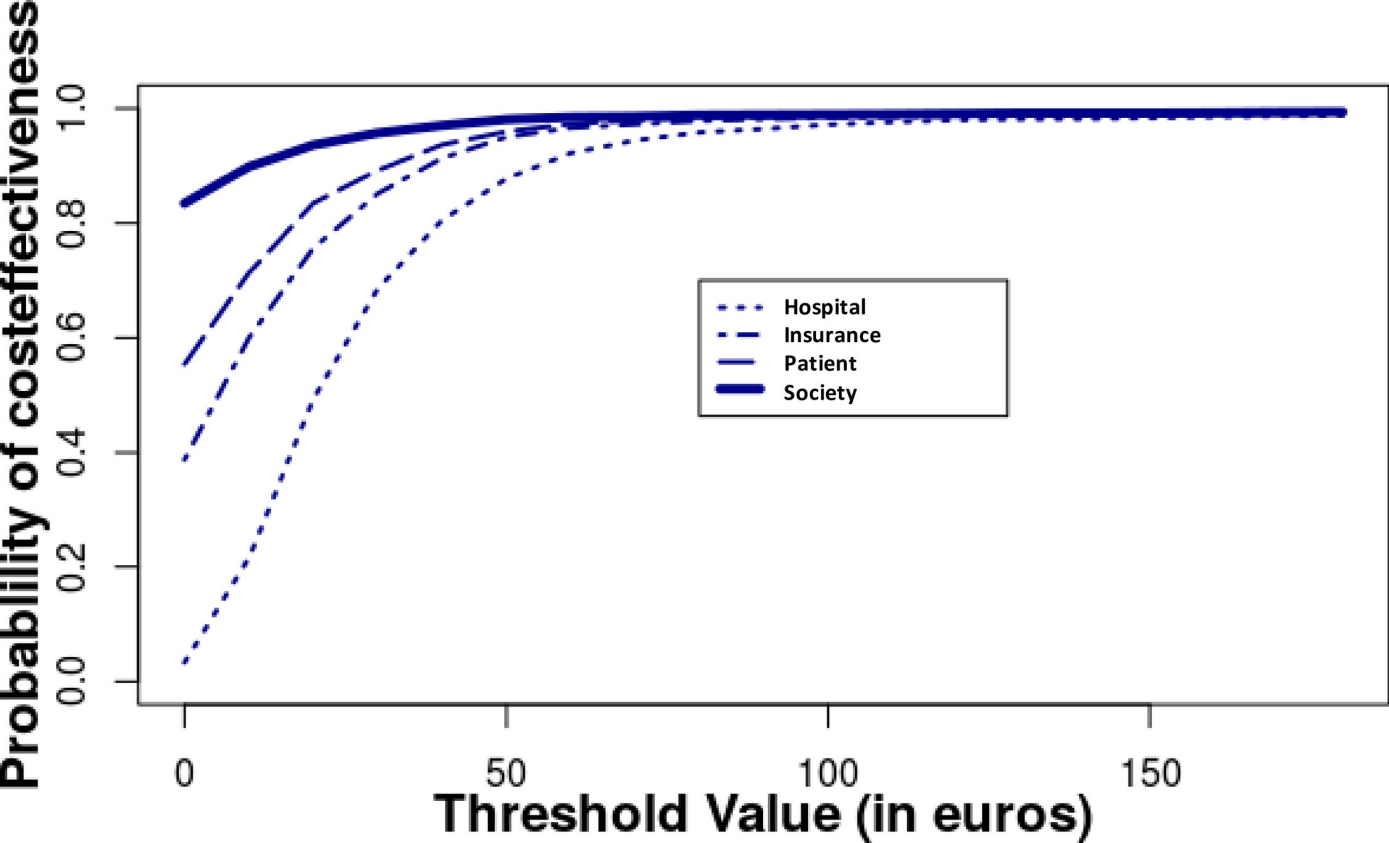
Acceptability curve (price for 1 day with pain NRS ≤ 4/10) from the societal perspective. Fig 3 shows the probability of the intervention being cost effective using the base case data for a range of cost effectiveness ceilings. Hospital: hospital costs only, Insurance: health insurance costs added, patient: costs at the patient’s charge added, society: costs related to absenteeism and presenteeism added.

### Sensitivity analyses

We ran three deterministic sensitivity analyses in addition to the bootstrap probabilistic analyses. For the first one, we choose as primary outcomes the proportion of days with pain NRS ≤ 4 defined as a self-assessed worst daily pain of 2/10 or lower. For the second run, we excluded extreme values. Only two patients in the acupuncture groups presented substantially high length of stay for hospitalizations. These patients were excluded in a sensitivity analysis. For the third analysis, we restricted costs estimation only to direct costs, as required by the French health authority. We did not identify drivers of uncertainty.

## Discussion

### Main findings

Pregnant women with pelvic girdle and low back pain treated with acupuncture in addition to routine care reported less pain and disability than those who received routine care alone. Acupuncture with routine care dominated (cheaper and more effective) routine care alone from the perspective of society and cost an additional €22 for each additional day with a pain score ≤ 4 from the perspective of the hospital. From the societal perspective, the higher direct healthcare costs of acupuncture were offset by lower indirect costs of absence from work.

### Strength and limitations

As for evaluating the effectiveness of acupuncture, the fact that the endpoint was measured up to delivery for both pain and disability adds to previous studies that focussed on short-term outcome[14–18]. Using the Oswestry score had the advantage of covering a reasonably wide spectrum of activities, including personal care, lifting, walking, sitting, standing, sex life, social life, and travelling[21]. Evaluating pain and disability based on self-assessment partly limited the bias related to healthcare providers not being blinded to the treatment. We chose not to use the EQ5D Instrument because we feared follow-up would be too short to detect a substantial QALY difference. Besides, the responsiveness of EQ5D might be suboptimal when back pain is considered[35].

The external validity of our results may be limited by a potential selection bias in the recruitment process. Pregnant women with low back pain who elected to participate may have been more psychologically prone to benefit from acupuncture than those who did not show up. We are not able to ascertain this bias. Regarding internal validity, the absence of placebo acupuncture precluded evaluation of the placebo effect of acupuncture, which may be substantial during pregnancy[18]. Because the evaluation of cost and effectiveness relied on a patient-completed logbook, recall bias is possible. Missing data required imputation, and some data may not be missing at random. To avoid the bias resulting from overrepresentation of some individuals, we used a Heckman sample selection model. Sample selection is a specific form of endogeneity, implying that we might have misidentified causal relationship between the outcome variables describing pain and disability, and explanatory variables such as the type of treatment. Actually, patients in the control group discontinued sending their follow up logbooks more often than did those in the acupuncture group, resulting into "incidental truncation" of data. To correct for this, we had to identify and integrate the selection mechanisms leading to truncation into our model. We used socioeconomic variables, such as education, socio-professional category, marital status, and parity to characterise those mechanisms. The underlying rationale is that such factors are likely to explain the selection, i.e. if patients completed or not their logbooks, but are unlikely to affect the outcome. Those variables were therefore used to perform imputation using the Heckman method. This aimed at predicting the likelihood of completing daily and correctly the logbook on health outcomes with a first-stage equation, using a generalized linear mixed-effects model and the chosen instruments. Then, we estimated the NRS pain and Oswestry scores using the first-stage equation's parameters. Imputed values were compared with a typical mixed-model imputation and found to be almost similar.

We choose not to exclude a “do-nothing” strategy in the control group, but to provide patients with information on physical activity and pregnancy belt. Drugs, physiotherapy, or other actions were prescribed by the physician or midwife in charge, in order to reflect usual practice.

The trial was stopped before we reached the planned number of cases, due to lack of funding. This is not likely to alter the interpretation of results, since we found a significant difference in outcome between the intervention group and controls.

### Interpretation

So far no cost effectiveness study was undertaken to evaluate acupuncture as treatment of low back pain during pregnancy. Previous studies in non-pregnant persons concluded to a modest health benefit for minor extra cost compared with usual care, from the point of view of the health care system[36] or of society at large[37]. A pilot paper argued that trials such as the one we report, would be welcomed by women and clinicians[38]. The short-term effect of acupuncture we observed was slightly lower than previously reported. This may be related to our offering a smaller number of acupuncture sessions than what was provided in previous studies^16^. Whether the effect of acupuncture results from needling *per se*, or from the attention and care given by a dedicated professional remains unclear.

## Conclusion

In addition to standard care, acupuncture was beneficial to pregnant women with PGLBP. Acupuncture represented an additional cost from the hospital point of view, but was a dominant strategy, i.e. cost saving and more effective, from the point of view of society at large, when both healthcare and non-healthcare costs were included These results could help public health decision-makers consider providing acupuncture for pelvic and low back pain in pregnancy. For example, hospitals could offer acupuncture for low back pain as routine practice, which would require specific funding. Alternatively, health insurance programs could reimburse acupuncture in specific indications.

## Supporting information

S1 File We completed the cheers checklist. (DOCX)

S2 File We completed the Consort checklist. (DOCX)

S3 File Study protocol in French. (PDF)

S1 Fig Cost effectiveness plane, outcome: Decrease in pain numerical rating scale between baseline and week 5 after inclusion. We performed 2,000 bootstrap replications of the cost effectiveness ratio. The outcome was the decrease in pain numerical rating scale between baseline and week 5 after inclusion, expressed as a difference between acupuncture and standard care. All costs were taken into account.
(TIF)

S2 Fig Cost effectiveness plane, outcome: Number of days with pain numerical rating scale ≤ 4/10 (hospital perspective). We performed 2,000 bootstrap replications of the cost effectiveness ratio. The outcome was the number of days with pain NRS ≤ 4/10 between inclusion and delivery expressed as a difference between acupuncture and standard care. Only hospital costs were taken into account. From a hospital perspective, acupuncture was more effective and more costly than routine care.
(TIF)

S3 Fig Cost effectiveness plane, outcome Cost effectiveness plane, outcome: Number of weeks with Oswestry score ≤ 20 after acupuncture. We performed 2,000 bootstrap replications of the cost effectiveness ratio. The outcome was the number of weeks with pain Oswestry score ≤ 20 between inclusion and delivery, expressed as a difference between acupuncture and standard care. All costs were taken into account. Acupuncture was more effective and less costly than standard care.
(TIF)

S1 Table Traditional Chinese Medicine diagnosis criteria. Traditional Chinese medicine diagnosis was based on clinical examination at inclusion. It was the first step of acupuncture treatment.
(DOC)

S2 Table Acupoints. Baseline points were applied to all patients in the acupuncture group. Other points were used according to pain location and to traditional Chinese medicine (TCM) diagnosis.
(DOC)

S3 Table Unit costs of resources in euros. We calculated costs from the perspective of the health care system (health insurance plus hospital costs), the patient (costs at the patient’s charge), and the employer (sick leave and presenteeism).
(DOC)

S4 Table Baseline characteristics, social background. Social back ground was similar in the acupuncture group and in the control group.
(DOC)

S5 Table Perinatal outcome. There was no significant difference between the acupuncture and the control group.
(DOC)

S6 Table Utilisation of healthcare resources and cost for different payers. We calculated costs from the perspective of the health care system (health insurance plus hospital costs), the patient (costs at the patient’s charge), and the employer (sick leave and presenteeism).
(DOC)

## References

[1] Berg G, Hammar M, Möller-Nielsen J, Lindén U, Thorblad J. Low back pain during pregnancy. Obstet Gynecol. 1988;71: 71–75. 2962023

[2] Wu WH, Meijer OG, Uegaki K, Mens JMA, van Dieën JH, Wuisman PIJM, et al Pregnancy-related pelvic girdle pain (PPP), I: Terminology, clinical presentation, and prevalence. Eur Spine J Off Publ Eur Spine Soc Eur Spinal Deform Soc Eur Sect Cerv Spine Res Soc. 2004;13: 575–589. 10.1007/s00586-003-0615-y 15338362 PMC3476662

[3] Wang S-M, Dezinno P, Maranets I, Berman MR, Caldwell-Andrews AA, Kain ZN. Low back pain during pregnancy: prevalence, risk factors, and outcomes. Obstet Gynecol. 2004;104: 65–70. 10.1097/01.AOG.0000129403.54061.0e 15229002

[4] Ostgaard HC, Andersson GB, Karlsson K. Prevalence of back pain in pregnancy. Spine. 1991;16: 549–552. 1828912 10.1097/00007632-199105000-00011

[5] Vermani E, Mittal R, Weeks A. Pelvic girdle pain and low back pain in pregnancy: a review. Pain Pract Off J World Inst Pain. 2010;10: 60–71. 10.1111/j.1533-2500.2009.00327.x 19863747

[6] Vleeming A, Albert HB, Ostgaard HC, Sturesson B, Stuge B. European guidelines for the diagnosis and treatment of pelvic girdle pain. Eur Spine J Off Publ Eur Spine Soc Eur Spinal Deform Soc Eur Sect Cerv Spine Res Soc. 2008;17: 794–819. 10.1007/s00586-008-0602-4 18259783 PMC2518998

[7] Close C, Sinclair M, Liddle SD, Madden E, McCullough JEM, Hughes C. A systematic review investigating the effectiveness of Complementary and Alternative Medicine (CAM) for the management of low back and/or pelvic pain (LBPP) in pregnancy. J Adv Nurs. 2014;70: 1702–1716. 10.1111/jan.12360 24605910

[8] Albert H, Godskesen M, Westergaard J. Evaluation of clinical tests used in classification procedures in pregnancy-related pelvic joint pain. Eur Spine J Off Publ Eur Spine Soc Eur Spinal Deform Soc Eur Sect Cerv Spine Res Soc. 2000;9: 161–166. 10.1007/s005860050228 PMC3611366 10823434

[9] Norén L, Ostgaard S, Johansson G, Ostgaard HC. Lumbar back and posterior pelvic pain during pregnancy: a 3-year follow-up. Eur Spine J Off Publ Eur Spine Soc Eur Spinal Deform Soc Eur Sect Cerv Spine Res Soc. 2002;11: 267–271. 10.1007/s00586-001-0357-7 12107796 PMC3610523

[10] Olsson C, Nilsson-Wikmar L. Health-related quality of life and physical ability among pregnant women with and without back pain in late pregnancy. Acta Obstet Gynecol Scand. 2004;83: 351–357. 15005782

[11] Ostgaard HC. Assessment and treatment of low back pain in working pregnant women. Semin Perinatol. 1996;20: 61–69. 8899915 10.1016/s0146-0005(96)80058-9

[12] Gutke A, Lundberg M, Östgaard HC, Öberg B. Impact of postpartum lumbopelvic pain on disability, pain intensity, health-related quality of life, activity level, kinesiophobia, and depressive symptoms. Eur Spine J Off Publ Eur Spine Soc Eur Spinal Deform Soc Eur Sect Cerv Spine Res Soc. 2011;20: 440–448. 10.1007/s00586-010-1487-6 20593205 PMC3048223

[13] The National Institute for Health and Care Excellence (NICE—UK). Low back pain (early management) [Internet]. 2009. Available: https://pathways.nice.org.uk/pathways/low-back-pain-early-management

[14] Wedenberg K, Moen B, Norling A. A prospective randomized study comparing acupuncture with physiotherapy for low-back and pelvic pain in pregnancy. Acta Obstet Gynecol Scand. 2000;79: 331–335. 10830757

[15] Guerreiro da Silva JB, Nakamura MU, Cordeiro JA, Kulay L. Acupuncture for low back pain in pregnancy—a prospective, quasi-randomised, controlled study. Acupunct Med J Br Med Acupunct Soc. 2004;22: 60–67. 10.1136/aim.22.2.60 15253580

[16] Elden H, Ladfors L, Olsen MF, Ostgaard H-C, Hagberg H. Effects of acupuncture and stabilising exercises as adjunct to standard treatment in pregnant women with pelvic girdle pain: randomised single blind controlled trial. BMJ. 2005;330: 761 10.1136/bmj.38397.507014.E0 15778231 PMC555879

[17] Lund I, Lundeberg T, Lönnberg L, Svensson E. Decrease of pregnant women’s pelvic pain after acupuncture: a randomized controlled single-blind study. Acta Obstet Gynecol Scand. 2006;85: 12–19. 16521674 10.1080/00016340500317153

[18] Elden H, Fagevik-Olsen M, Ostgaard H-C, Stener-Victorin E, Hagberg H. Acupuncture as an adjunct to standard treatment for pelvic girdle pain in pregnant women: randomised double-blinded controlled trial comparing acupuncture with non-penetrating sham acupuncture. BJOG Int J Obstet Gynaecol. 2008;115: 1655–1668. 10.1111/j.1471-0528.2008.01904.x 18947338

[19] Wang S-M, Dezinno P, Lin EC, Lin H, Yue JJ, Berman MR, et al Auricular acupuncture as a treatment for pregnant women who have low back and posterior pelvic pain: a pilot study. Am J Obstet Gynecol. 2009;201: 271.e1-9 10.1016/j.ajog.2009.04.028 19560110 PMC2768290

[20] Kvorning N, Holmberg C, Grennert L, Aberg A, Akeson J. Acupuncture relieves pelvic and low-back pain in late pregnancy. Acta Obstet Gynecol Scand. 2004;83: 246–250. 14995919 10.1111/j.0001-6349.2004.0215.x

[21] Ostelo RWJG Deyo RA, Stratford P, Waddell G, Croft P, Von Korff M, et al Interpreting change scores for pain and functional status in low back pain: towards international consensus regarding minimal important change. Spine. 2008;33: 90–94. 10.1097/BRS.0b013e31815e3a10 18165753

[22] Husereau D, Drummond M, Petrou S, Carswell C, Moher D, Greenberg D, et al Consolidated Health Economic Evaluation Reporting Standards (CHEERS)—explanation and elaboration: a report of the ISPOR Health Economic Evaluation Publication Guidelines Good Reporting Practices Task Force. Value Health J Int Soc Pharmacoeconomics Outcomes Res. 2013;16: 231–250. 10.1016/j.jval.2013.02.002 23538175

[23] ATIH. ENC MCO 2017 https://www.atih.sante.fr/etudes-nationales-de-couts-sanitaires-enc/presentation.

[24] Assurance Maladie. https://www.ameli.fr [Internet].

[25] INSEE. https://www.insee.fr/fr/statistiques/2021266.

[26] Dørheim SK, Bjorvatn B, Eberhard-Gran M. Sick leave during pregnancy: a longitudinal study of rates and risk factors in a Norwegian population. BJOG Int J Obstet Gynaecol. 2013;120: 521–530. 10.1111/1471-0528.12035 23130975

[27] Gatrell CJ. “I”m a bad mum’: pregnant presenteeism and poor health at work. Soc Sci Med 1982. 2011;72: 478–485. 10.1016/j.socscimed.2010.11.020 21194818

[28] Briggs AH, O’Brien BJ. The death of cost-minimization analysis? Health Econ. 2001;10: 179–184. 10.1002/hec.584 11252048

[29] Robinson R. Economic evaluation and health care. What does it mean? BMJ. 1993;307: 670–673. 8401057 10.1136/bmj.307.6905.670 PMC1678982

[30] Drummond MF, Sculpher MJ, Torrance GW, O’Brien BJ, Stoddart GL. Methods for the Economic Evaluation of Health Care Programmes. 3 edition Oxford; New York: Oxford University Press; 2005.

[31] Heckman J.J. Sample Selection Bias as a Specification Error. Econometrica 47,153–161. 1979.

[32] Clark SJ, Houle B. Validation, replication, and sensitivity testing of Heckman-type selection models to adjust estimates of HIV prevalence. PloS One. 2014;9: e112563 10.1371/journal.pone.0112563 25402333 PMC4234405

[33] Galimard J-E, Chevret S, Protopopescu C, Resche-Rigon M. A multiple imputation approach for MNAR mechanisms compatible with Heckman’s model. Stat Med. 2016;35: 2907–2920. 10.1002/sim.6902 26893215

[34] Puhani. The Heckman Correction for Sample Selection and Its Critique. Journal of Economic Surveys. 2002 Available: http://onlinelibrary.wiley.com/doi/10.1111/1467-6419.00104/pdf. Accessed 5 Oct 2016.

[35] Whynes DK, McCahon RA, Ravenscroft A, Hodgkinson V, Evley R, Hardman JG. Responsiveness of the EQ-5D health-related quality-of-life instrument in assessing low back pain. Value Health J Int Soc Pharmacoeconomics Outcomes Res. 2013;16: 124–132. 10.1016/j.jval.2012.09.003 23337223

[36] Ratcliffe J, Thomas KJ, MacPherson H, Brazier J. A randomised controlled trial of acupuncture care for persistent low back pain: cost effectiveness analysis. BMJ. 2006;333: 626 10.1136/bmj.38932.806134.7C 16980315 PMC1570795

[37] Witt CM, Jena S, Selim D, Brinkhaus B, Reinhold T, Wruck K, et al Pragmatic randomized trial evaluating the clinical and economic effectiveness of acupuncture for chronic low back pain. Am J Epidemiol. 2006;164: 487–496. 10.1093/aje/kwj224 16798792

[38] Foster NE, Bishop A, Bartlam B, Ogollah R, Barlas P, Holden M, et al Evaluating Acupuncture and Standard carE for pregnant women with Back pain (EASE Back): a feasibility study and pilot randomised trial. Health Technol Assess Winch Engl. 2016;20: 1–236. 10.3310/hta20330 27133814 PMC4867424

