# Supplementary material for: Cost-effectiveness of acupuncture versus standard care for pelvic and low back pain in pregnancy: A randomized controlled trial
Source: PLoS One. 2019 Apr 22;14(4):e0214195. doi: 10.1371/journal.pone.0214195 (PMC6476478; doi:10.1371/journal.pone.0214195)
Supplement: S3 File — (PDF) [file pone.0214195.s003.pdf]

GAME
Acupuncture pour les douleurs lombaires et de la ceinture pelvienne pendant la grossesse : étude médico-économique

PROTOCOLE DE
RECHERCHE BIOMEDICALE

Version N°2 du 23/07/2012
Code projet : P111011
EudraCT : 2012-A00240-43

Investigateur Coordonnateur:

Pr. Marc DOMMERGUES, Service de Gynécologie Obstétrique Groupe Hospitalier Pitié Salpêtrière, 47-83 Bd de l'Hôpital 75651 Paris Cedex 13 – France

AP-HP, Paris, et Université Pierre et Marie Curie, Paris VI

Téléphone : 01 42 17 77 01 / 06 08 48 69 25 Télécopie : 01 42 17 77 02

Courriel : marc.dommergues@psl.aphp.fr

Promoteur :

Assistance Publique - Hôpitaux de Paris,

DRCD, Carré Historique de l'Hôpital Saint Louis

1 avenue Claude Vellefaux 75475 PARIS Cedex 10

Chef de projet : Ludovic DYEN, Téléphone : 01 44 84 17 43 Télécopie : 01 44 84 17 01

Courriel : ludovic.dyen@sls.aphp.fr

URC : Pitié Salpêtrière - Charles-Foix

Pr. Alain MALLET

47-83 Bd de l'Hôpital 75651 Paris Cedex 13 – France

Téléphone : 01 42 16 05 05 Télécopie : 01 42 16 05 13

CEC : Karine MARTIN, Téléphone : 01 42 16 24 43 Télécopie : 01 42 16 24 40
Courriel : karine.martin@psl.aphp.fr

ARC : Stéphanie LALLAURET, Téléphone : 01 42 16 24 37 Télécopie : 01 42 16 24 40
Courriel : stephanie.lallauret@psl.aphp.fr

Page de SIGNATURE D'UN PROTOCOLE de recherche biomédicale par l'investigateur coordonnateur et le représentant du promoteur

Code de la Recherche biomédicale : P111011

EudraCT : 2012-A00240-43.

*Titre : «**GAME : Acupuncture pour douleurs lombaires et de la ceinture pelvienne pendant la grossesse : étude médico-économique**»*

Version N° 2.0 du 23/07/2012

<u>L'investigateur coordonnateur</u> :
Pr. Marc DOMMERGUES

Service de Gynécologie Obstétrique
Groupe Hospitalier Pitié Salpêtrière,
47-83 Bd de l'Hôpital 75651 Paris Cedex 13 –

Date : ………/………/ 2012

Signature :

<u>Pour le promoteur :</u>
Christophe MISSE

Assistance Publique – Hôpitaux de Paris
Département de la Recherche Clinique et du Développement
Hôpital Saint Louis
75010 PARIS

Date : ……………/………/2012

Signature :

NB : cette version correspond au texte du protocole et annexes adressés au CPP Pitié-Salpêtrière – Ile-de-France VI ayant obtenue l'autorisation initiale le 27/06/2012 et à l'autorité compétente respectivement pour avis et demande d'autorisation, reçus le 22/05/2012.

LISTE DES CENTRES et investigateur principal de chaque centre

Centre procédant à l'inclusion des patientes

#	Centres	Services - Adresses	Investigateurs Principaux
**001**	**Service de Gynécologie Obstétrique**<br>**GH Pitié Salpêtrière**	**Service de Gynécologie Obstétrique**<br>Groupe Hospitalier Pitié-Salpêtrière<br>47-83 Bd de l'Hôpital<br>75651 Paris Cedex 13	**Pr. Marc Dommergues**<br>marc.dommergues@psl.aphp.fr
**002**	**CIC Paris-Est**<br>**GH Pitié Salpêtrière**	**Centre d'Investigation Clinique Paris-Est**<br>Groupe Hospitalier Pitié-Salpêtrière<br>47-83 Bd de l'Hôpital<br>75651 Paris Cedex 13	**Dr Beny Charbit**<br>beny.charbit@sat.aphp.fr

Centres procédant au screening des patientes

#	Centres	Services - Adresses	Investigateurs Associés
**501**	**Hôpital du Kremlin Bicêtre**	**Service de Gynécologie Obstétrique**<br>Hôpital Bicêtre<br>78 rue du Général Leclerc<br>94270 Le Kremlin Bicêtre	**Pr. Hervé Fernandez**<br>herve.fernandez@bct.aphp.fr
**502**	**Hôpital Necker**	**Service de Gynécologie Obstétrique**<br>Hôpital Necker Enfants Malades<br>149 rue de Sèvres<br>75015 Paris	**Pr. Yves Ville**<br>Ville.yves@gmail.com
**503**	**Clinique Jeanne d'Arc**	**Service de Gynécologie Obstétrique**<br>Clinique Jeanne d'Arc<br>11 rue Ponscarme<br>75013 Paris	**Dr Jean-Simon Arfi**<br>sjarfi@wanadoo.fr
**504**	**Hôpital Trousseau**	**Service de Gynécologie-Obstétrique**<br>Hôpital Trousseau<br>26 av du Dr. Arnold NETTER<br>75012 Paris	**Pr. Jean-Louis Benifla**<br>jl.benifla@trs.aphp.fr
**505**	**Hôpital des Diaconesses**	**Service de Gynécologie Obstétrique**<br>Groupe Hospitalier Diaconesses<br>Croix Saint Simon<br>18 rue du sergent Bauchat<br>75012 Paris	**Dr Thierry Harvey**<br>tharvey@hopital-dcss.org
**506**	**Hôpital Tenon**	**Service de Gynécologie Obstétrique**<br>Hôpital Tenon<br>4 rue de la Chine<br>75020 Paris	**Pr. Emile DARAI**<br>emile.darai@tnn.aphp.fr
**507**	**Hôpital Pitié Salpêtrière**	**Service de Gynécologie Obstétrique**<br>Groupe Hospitalier Pitié-Salpêtrière<br>47-83 Bd de l'Hôpital<br>75013 Paris	**Pr. Marc Dommergues**<br>marc.dommergues@psl.aphp.fr

INVESTIGATEURS ASSOCIES	POLE D'ACTIVITE ou SERVICE	ETABLISSEMENTS DE SANTE
Pr Isabelle Durand Zaleski, PUPH	URC-ECO	AP-HP
Dr Beny Charbit, MCUPH	CIC	GH Pitié Salpêtrière
Mme Stéphanie Nicolian	Sce Gynécologie - Obstétrique	GH Pitié Salpêtrière
Dr M. Bingkai Liu	Dép. Recherche Clinique	AP-HP
Dr A Couteaux	Centre de lutte contre la douleur	GH Pitié Salpêtrière

Plateaux techniques utilisés:

Consultation d'obstétrique de chaque service impliqué

CIC Paris-Est Pitié-Salpêtrière pour la réalisation de l'intervention « acupuncture ».

Méthodologistes :

Pr. Alain Mallet (URC GH Pitié Salpêtrière)

Pr. Isabelle Durand Zaleski (URC ECO, APHP, Paris)

Sommaire

LISTE DES CENTRES et investigateur principal de chaque centre 3

Résumé 7

Abréviations 8

II. OBJECTIFS DE LA RECHERCHE 11

III. CONCEPTION DE LA RECHERCHE 12

III.1 Critères d'évaluation 12

III.1.1 Critères principaux 12

III.1.2 Critères secondaires 12

III.2 Description de la méthodologie 13

III.2.1 Recrutement des patientes : présélection 13

III.2.2 Critères d'inclusion 13

III.2.3 Critères de non-inclusion 14

III.2.4 Inclusion et randomisation 14

III.2.5 Interventions 14

III.2.6 Suivi après inclusion 21

III.2.7 Nombre de sujets nécessaire 21

III.3 Faisabilité 22

III.3.1 Compétence des investigateurs en acupuncture 22

III.3.2 Locaux dédiés et logistique 22

III.3.3 Capacité à recruter des patientes 22

III.3.4 Motivation des femmes enceintes 22

III.4 Mesures prises pour éviter les biais 23

III.5 Données recueillies 23

III.5.1 Informations recueillies à l'inclusion 23

III.5.2 Informations recueillies lors du suivi 24

III.5.3 Protocole d'acupuncture 25

III.5.4 Perdues de vue et abandon du suivi 25

III.5.5 Rupture de protocole 25

III.5.6 Informations recueillies après l'accouchement 25

III.6 Distinction soin / recherche 26

IV. ANALYSE STATISTIQUE 26

IV.1 Analyses principales 26

IV.2 Analyses secondaires 26

IV.3 Nombre de sujets nécessaires 27

IV.4 Analyse économique 28

V. RESULTATS ATTENDUS ET PERSPECTIVES. 30

V.1 Résultats attendus 30

V.2 Critère de jugement principal 31

V.3 Critères secondaires 31

V.4 Perspectives 31

VI. TRAITEMENT ADMINISTRE AUX PERSONNES QUI SE PRETENT A LA RECHERCHE 31

VII. EVALUATION DE LA SECURITE 31

VII.1 Description des paramètres d'évaluation de la sécurité 32

VII.2 Méthodes et calendrier prévus pour mesurer, recueillir et analyser les paramètres d'évaluation de la sécurité 32

VII.2.1 Comité de pilotage 32

VII.2.2 Comité de surveillance indépendant 33

VII.3 Procédures mises en place en vue de l'enregistrement et de la notification des évènements indésirables 33

VII.3.1 Evènements indésirables non graves 33

VII.3.2 Evènements indésirables graves (EIG) 33

VII.3.3 Déclaration des évènements indésirables graves aux Autorités de Santé 34

VII.4 Dispositions à prendre en vue d'assurer la sécurité en cas de défaillance du dispositif médical y compris en cas de dysfonctionnement isolé du dispositif sans retentissement clinique ainsi qu'en cas de mauvaise utilisation 35
VII.5 Le protocole décrit les risques possibles et les effets indésirables prévisibles sur la base des données disponibles 35
VIII. DROIT D'ACCES AUX DONNEES ET DOCUMENTS SOURCE 35
IX. CONTROLE ET ASSURANCE DE LA QUALITE 36
IX.1 Procédures de monitoring 36
IX.2 Transcription des données dans le cahier d'observation 36
X. CONSIDERATIONS LEGALES ET ETHIQUES 37
X.1 Demande d'autorisation auprès de l'ANSM 37
X.2 Demande d'avis au Comité de Protection des Personnes 37
X.3 Modifications 38
X.4 Déclaration CNIL 38
X.5 Note d'information et Consentement éclairé 39
X.6 Rapport final de la recherche 39
XI. TRAITEMENT DES DONNEES ET CONSERVATION DES DOCUMENTS ET DES DONNEES RELATIVES A LA RECHERCHE 39
XII. ASSURANCE ET ENGAGEMENT SCIENTIFIQUE 40
XII.1 Assurance 40
XII.2 Engagement de responsabilités 40
XIII. REGLES RELATIVES A LA PUBLICATION 40
XIV. BIBLIOGRAPHIE 41
XV. Liste des annexes 43
XV.1 Annexe 1. Score d'incapacité fonctionnelle d'Oswestry 43
XV.2 Annexe 2. Grille de Classification des Evénements Indésirables pour une Recherche Biomédicale portant sur un dispositif médical 46

Résumé

Titre	**Acupuncture pour douleurs lombaires et de la ceinture pelvienne pendant la grossesse : étude médico-économique**
**Etat de la question**	En cours de grossesse, les douleurs lombaires et de la ceinture pelvienne (DLCP) sont fréquentes (# 30%), régressent en général à distance de l'accouchement, et sont invalidantes chez 5 à 10 % des femmes. Les traitements habituels, basés sur les antalgiques et l'hygiène de vie sont d'une efficacité limitée. L'adjonction de l'acupuncture en complément des traitements habituels pourrait améliorer la qualité de la vie. Quatre essais randomisés contrôlés ont montré que l'acupuncture, pratiquée en complément des traitements habituels, réduisait la douleur de 50 à 60%, à court terme, cet effet étant supérieur à celui des traitements habituels seuls (réduction de la douleur de 10 à 20%). On ignore l'impact de l'acupuncture sur la douleur évaluée à moyen terme, tout au long de la grossesse.
**Objectif**	Déterminer l'impact médical et économique d'une offre de soin d'acupuncture pour les DLCP grossesse
**Critères de sélection**	**- Critères d'inclusion** : Grossesse monofoetale entre 16 et 35 semaines d'aménorrhée, DLCP, intensité de la douleur > 4/10.<br><br>**- Critères de non-inclusion** : Menace d'accouchement prématuré, retard de croissance avéré < 5ème percentile, pré-éclampsie, maladie rhumatologique chronique, sciatique typique isolée, âge maternel < 18 ans, absence de couverture sociale
**Nombre de sujets**	300
**Durée de la recherche**	3 ans, 2 ans ½ d'inclusion et 6 mois maximum de participation
**Durée de participation de chaque patiente**	Entre inclusion et accouchement, soit 5 à 26 semaines
**Méthodologie:**	- Essai thérapeutique multicentrique randomisé contre traitement de référence, ouvert, en groupes parallèles.<br>- Intervention : 5 séances d'acupuncture en 4 semaines puis séances à la demande, associées au traitement habituel. Groupe témoin : traitement habituel.<br>- Unité d'analyse : la grossesse.<br>- Suivi : carnet de bord tenu par la patiente pendant toute la grossesse et analyse du dossier obstétrical après l'accouchement
**Critères d'évaluation principaux**	Score de douleur 5 semaines après l'inclusion. Nombre et % de jours avec douleur maximale quotidienne ≤4/10 entre l'inclusion et l'accouchement
**Critères d'évaluation secondaires**	- Score de limitation fonctionnelle moyen entre inclusion et accouchement (Oswestry)<br>- Coût (1) des soins de santé depuis l'inclusion, et (2) des conséquences de la limitation fonctionnelle, notamment arrêt de travail avant congé maternité, transports médicalisés<br>- Recours aux professionnels de santé (médecins, sage femmes, kinésithérapeutes, médecines complémentaires…)<br>- Nombre de jours de consommation d'antalgiques<br>- Nombre de séance d'acupuncture et observance du traitement<br>- Taux d'échec à 4 semaines (score de douleur ≥ à celui à l'inclusion)<br>- Indicateurs de morbidité obstétricale et périnatale: taux de césarienne, de prématurité, de retard de croissance intra utérin, d'admission en néonatologie, score d'Apgar
**Résultats attendus**	Dans le groupe acupuncture on attend par rapport au groupe témoin :<br>- Réduction de 20% de la douleur (score et jours avec douleur >4), et de l'incapacité fonctionnelle<br>- Pas de modification des indicateurs de morbidité obstétricale et périnatale
**Perspectives**	Contribuer à quantifier les bénéfices cliniques et les coûts résultant d'une offre de soins hospitalière d'acupuncture pour les femmes enceintes
**Mots Clés**	Acupuncture, médecine complémentaire, douleur, grossesse, qualité de la vie, invalidité

Abréviations

ARC Attaché de Recherche Clinique

CIC Centre d’Investigation Clinique

DLCP Douleurs lombaires et de la ceinture pelvienne

EVA Echelle Visuelle Analogique

GHPS Groupe Hospitalier Pitié Salpêtrière

MTC Médecine Traditionnelle Chinoise

sa Semaines d’aménorrhée

TEC Technicien d’Etude Clinique

URC Unité de Recherche Clinique

I. JUSTIFICATION SCIENTIFIQUE DE LA RECHERCHE

Les douleurs lombaires et de la ceinture pelvienne (DLCP) sont fréquentes en cours de grossesse. Décrites en 1948 par Maurice Lacomme comme d'origine osteo-musculaire (Malinas 1993), elles concernent 5 à 30% des femmes enceintes selon le seuil d'intensité douloureuse considéré, et la définition clinique utilisée (Timsit 2004, Vermani 2010). La physiopathologie des DLCP est complexe et multifactorielle (Waynberger et al. 2005).
Le diagnostic repose sur l'examen clinique : analyse des plaintes fonctionnelles, tests simples de provocation de la douleur, exclusion des diagnostics différentiels graves (Wu 2004).
Ces douleurs sont rapportées par les patientes comme siégeant au niveau du bassin, du bas du dos, de l'aine, du pubis, des cuisses. Elles sont favorisées par la position debout et l'activité physique.
Les douleurs de la ceinture pelvienne et les douleurs lombaires basses sont souvent intriquées et analysées comme une seule entité (Vermani 2010). Elles sont fréquemment associées à des douleurs lombaires hautes et dorsales. Leur intensité varie au cours de la journée, plus intense le soir que le matin (Hansen 1999, Lile 2003, Elden 2005, Vermani 2010). Elles peuvent régresser dans les mois qui suivent l'accouchement, mais ont un impact négatif sur la qualité de la vie après l'accouchement (Ostgard 1996, Gutke A. et al.2010). Environ 20 % des femmes présentant des DLCP de la grossesse souffrent encore 3 ans après (Noren et al. 2002)
Même si elles ne sont pas associées à une augmentation de la morbidité néonatale, et régressent spontanément à distance de la grossesse, les DLCP ont un impact substantiel sur la qualité de vie et engendrent un certain degré d'incapacité fonctionnelle ou professionnelle (Noren 1997 et 2002, Sydsjo 1998, Olsson et al 2004, Robinson et al. 2006 et 2010). Mosheni-Bandpei rapporte un score d'incapacité moyen de 34 % (score d'Oswestry) en cas de DLCP (Mohseni-Bandpei MA. et al. 2009). Wedenberg K et al rapportent des indices d'incapacité moyens de 5/8 à 7/8 pour des activités telles que monter un escalier, s'asseoir, courir, porter des objets (Wedenberg K. et al. 2000.)

Les professionnels encouragent les femmes enceintes à supporter ces « maux naturels et sans danger» de la grossesse, et proposent des stratégies de prise en charge diverses : repos, conseils de postures et de mode de vie, port d'une ceinture de grossesse, antalgiques, kinésithérapie, exercices en piscine, ostéopathie, yoga, relaxation, aromathérapie, massages, compresses chaudes ou froides, acupuncture.
A partir d'essais cliniques contrôlés randomisés comparant chaque technique à une prise en charge dite standard, une revue de la Cochrane Database concluait à l'efficacité possible de l'acupuncture, des exercices de stabilisation du dos, des étirements (Pennick et Young, 2008).
Trois essais thérapeutiques randomisés (Wendenberg 2000, Kworning 2004, Elden 2005), et une étude contrôlée (Guerreiro da Silva, 2004) ont montré l'efficacité de l'acupuncture en association avec le traitement dit habituel (**Tableau 1**). A titre d'exemple, dans l'étude de Elden (2005), le critère principal était l'auto évaluation de la douleur provoquée par les mouvements, en fin de journée, au moyen d'une échelle visuelle analogique de 0 à 100. Cet essai était centré sur les douleurs de la ceinture pelvienne. Le traitement habituel seul (repos, conseils, exercices prescrits à faire à la maison) aboutissait à une faible diminution de la douleur (EVA moyenne 63 avant traitement, EVA moyenne 59 après traitement). Le traitement dit habituel associé à des exercices supplémentaires, contrôlés de façon hebdomadaire

par un kinésithérapeute (durée totale des séances : 6 heures) faisait diminuer la douleur d'une EVA moyenne de 60 avant traitement à 45 après. L'acupuncture (12 séances sur 6 semaines) associée au traitement habituel faisait diminuer la douleur d'une EVA moyenne de 65 avant traitement à 31 après. Cette diminution de la douleur était observée dès la première semaine de traitement. Des résultats similaires ont été retrouvés dans les autres essais. Dans l'étude de Wendenberg, une amélioration moyenne d'environ 30% des indices d'invalidité était notée dans le groupe acupuncture, contre une absence d'amélioration dans le groupe kinésithérapie.

Les données publiées suggèrent que l'acupuncture est un traitement complémentaire efficace pour soulager à court terme la douleur et l'incapacité fonctionnelle des femmes enceintes souffrant de douleurs de la ceinture pelvienne ou de douleurs lombaires.

On ignore si l'acupuncture agit directement sur la douleur, ou par un effet placebo, lié par exemple à l'impact psychologique d'un traitement personnalisé avec actes de soin répétés (Smith, 2009). Cette question physiopathologique est difficile à résoudre, notamment du fait des controverses sur les techniques d'acupuncture placebo : les stimulations par insertion superficielle des aiguilles (Lund et al. 2006), ou par stimulation cutanée sans pénétration pourraient avoir une action propre (Elden, 2008).

Il n'existe pas d'étude de coût des prises en charges médicales par acupuncture pendant la grossesse.

Tableau 1. Essais thérapeutiques randomisés et étude contrôlée évaluant l'acupuncture dans les DLCP de la grossesse

Auteur (pays)	Année	Traités/Témoins	Témoins	Diminution de la douleur (acupuncture / témoin)
Wedenberg (Suède)	2000	30 vs 30	Kinésithérapie	60% / 31% (baisse du score de douleur)
Guerreiro da Silva (Brésil)	2004	27 vs 34	Traitement « standard »	78% / 14 % (nb de patientes soulagées)
Kworning (Suède)	2004	37 vs 35	Pas de recommandation spécifique	60% / 14% (baisse du score de douleur)
Elden (Suède)	2005	130 vs 125+131	Traitement standard ou traitement renforcé (kinésithérapie)	52% / 8% et 26% (baisse du score de douleur)
Lund (Suède)	2006	25 vs 22	Acupuncture superficielle	NS
Elden (Suède)	2008	58 vs 57	Acupuncture non pénétrante	45%/40% (baisse du score de douleur : NS)<br><br>Index d'incapacité meilleur dans le groupe acupuncture pénétrante (44) que dans le groupe témoin (55)

En résumé, on sait que l'acupuncture est efficace à court terme sur les douleurs lombaires basses et de la ceinture pelvienne de la grossesse. Le mécanisme d'action est mal connu.

On ignore quel est l'effet de l'acupuncture sur la douleur et la fonction à moyen terme, tout au long de la grossesse. On ignore également quel serait son coût dans notre système de santé.

Cette question est d'actualité : jusqu'à récemment, l'acupuncture était utilisée pendant la grossesse par un nombre restreint de professionnels, souvent en dehors du milieu hospitalier. Le nombre croissant d'essais contrôlés suggérant l'efficacité de l'acupuncture pose le problème des modalités de sa diffusion, de son impact sur la qualité de la vie des femmes enceintes souffrant de douleurs invalidantes, des coûts induits, mais également des coûts évités par cette offre de soin.

C'est pourquoi nous nous proposons de déterminer l'impact d'une offre de soin d'acupuncture pour les DLCP de la grossesse. Cet impact sera évalué cliniquement par la réduction des douleurs et de l'incapacité fonctionnelle, et économiquement par la mesure des coûts directs et indirects, l'évaluation étant effectuée tout au long de la grossesse.

II. OBJECTIFS DE LA RECHERCHE

Objectif principal :

- Confirmer l'efficacité de l'acupuncture sur la douleur pelvienne et lombaire de la grossesse après 4 semaines de traitement, et évaluer son efficacité tout au long de la grossesse.

Objectifs secondaires :

- Confirmer l'efficacité de l'acupuncture sur l'incapacité fonctionnelle après 4 semaines de traitement, et évaluer son efficacité tout au long de la grossesse.
- Comparer le coût de la prise en charge avec ou sans acupuncture, du point de vue du système de santé et du point de vue des patientes.
- Déterminer, dans chaque bras, le recours des femmes aux antalgiques, aux soins médicaux, aux médecines complémentaires.

III. CONCEPTION DE LA RECHERCHE

III.1 Critères d'évaluation

III.1.1 Critères principaux

- Evaluation de la douleur par auto-questionnaire quotidien, rempli de l'inclusion à l'accouchement (carnet de bord), au moyen d'une échelle numérique du type :

Selon vous quel chiffre décrit le mieux la douleur la plus intense que vous avez ressentie ce jour ? (Entourez le chiffre qui correspond le mieux)

Pas de douleur	0	1	2	3	4	5	6	7	8	9	10	Douleur la plus intense

Deux critères sont obtenus à partir du carnet de bord :

- Douleur ressentie 5 semaines après l'inclusion (1 semaine après la dernière séance de la première série) (effet à court terme de l'acupuncture sur la douleur).

- Pourcentage de jours entre l'inclusion et l'accouchement avec douleur maximale > 4/10 (effet à moyen terme de l'acupuncture sur la douleur).

III.1.2 Critères secondaires

- Evaluation hebdomadaire de la limitation fonctionnelle entre l'inclusion et l'accouchement par le score d'Oswestry (Robinson HS. et al. 2010, Mohseni-Bandpei MA. et al. 2009, Gutke A. et al. 2006, Gutke A. et al. 2010, Vogler D. et al. 2008) (Annexe 1)

- Nombre de jours d'arrêt de travail et de congé maternité supplémentaire

- Nombre de jours avec prise d'antalgiques

- Nombre de jours avec consommation de plantes médicinales ou équivalent

- Nombre de séances de kinésithérapie, ostéopathie, acupuncture, relaxation, gymnastique, ou d'autres pratiques à visée antalgique

- Nombre de consultations médicales

- Nombre de transports médicalisés

- Taux d'échec thérapeutique 4 semaines révolues après l'inclusion (douleur supérieure ou égale à la douleur à l'inclusion)

- Issue de grossesse (indicateurs de morbidité maternelle et néonatale)

III.2 Description de la méthodologie

III.2.1 Recrutement des patientes : présélection

Les femmes enceintes seront recrutées via les consultations d'obstétrique des centres participant. Contribueront au recrutement des femmes enceintes dans l'essai : une campagne de sensibilisation des professionnels effectuant les consultations et des personnels d'accueil à la prise en compte de la douleur, des affiches informant de la possibilité d'entrer dans l'essai, la diffusion large d'un feuillet d'information sur l'essai dans les salles d'attente et lors des prises de rendez vous, une information via les sites internet des institutions.

En pratique, les femmes sont « pré-incluses » dans leur maternité d'origine de la manière la plus simple possible afin que le processus d'inclusion ne soit pas un frein à la participation effective des centres. La femme enceinte est présélectionnée par le professionnel, médecin ou sage femme, qui suit sa grossesse. Ce dernier informe la femme enceinte oralement et par 2 documents écrits (plaquette d'information sur l'essai et note d'information) comportant le numéro de téléphone du CIC de la Pitié Salpêtrière. Il remplit un formulaire de présélection, adressé par mail ou fax au CIC du Groupe Hospitalier Pitié Salpêtrière (GHPS).

Stéphanie Nicollian, (sage femme pratiquant l'acupuncture) sera informée par mail ou fax de la présélection et prendra contact par téléphone avec la femme enceinte pour lui donner un premier rendez-vous.

Certaines femmes ayant pris connaissance de l'étude par la plaquette d'information, l'affiche ou le site internet[1] pourront appeler directement le CIC.

III.2.2 Critères d'inclusion

1. Patiente majeure (au moins 18 ans)
2. Grossesse mono fœtale
3. Douleur de la ceinture pelvienne ou lombaire évoluant depuis au moins une semaine
4. Femme enceinte de 4 à 8 mois (soit ≥ à 16 et ≤ à 35 sa)
5. Intensité de la douleur > 4/10, sur une échelle de 0 (pas de douleur) à 10 (la pire des douleurs imaginables)
6. Formulaire de présélection signé par le professionnel qui suit la grossesse précisant l'absence de contre indication à l'acupuncture
7. Bonne compréhension de l'étude
8. Consentement éclairé signé
9. Bénéficiaire d'un régime de sécurité sociale ou ayant droit

---

[1] http://www.medecinechinoise.aphp.fr/index.php/recherche/projets-en-cours/game

III.2.3 Critères de non-inclusion

1. Menace d'accouchement prématuré, retard de croissance avéré < 5ème percentile, pré éclampsie
2. Maladie rhumatologique chronique pré existante à la grossesse
3. Sciatique typique isolée.

III.2.4 Inclusion et randomisation

Lors de la première visite au CIC Paris Est, la sage femme vérifie à nouveau les critères d'inclusion, de non inclusion, les antécédents de la femme enceinte, les caractéristiques de la grossesse, les caractéristiques de la douleur et de la limitation fonctionnelle. Après vérification des critères d'inclusion et de non inclusion, la sage-femme remet à la patiente la note d'information et le consentement éclairé à signer. Une fois le consentement éclairé signé, les patientes sont randomisées pour recevoir soit le traitement habituel seul, soit le traitement habituel associé à l'acupuncture. La randomisation sera centralisée, administrée par l'Unité de Recherche Clinique de la Pitié-Salpêtrière. L'outil de randomisation sera Randoweb, développé par l'URC et opérant sur le Web. La randomisation sera stratifiée par centre et effectuée par blocs cohérents avec le nombre de patientes à inclure.

Les patientes randomisées dans le bras « acupuncture » bénéficieront de leur première séance d'acupuncture le jour de cette première visite au GHPS. Les patientes randomisées dans le bras « traitement habituel » bénéficieront de la prise en charge habituelle à cette première séance.

Toutes les séances d'acupuncture auront lieu au CIC Paris Est, GHPS.

III.2.5 Interventions

Traitement habituel :

Le traitement habituel comprend au moins :

- Une information orale et écrite sur la physiopathologie des douleurs pelvienne de la grossesse.
- Une recommandation d'exercices à faire à la maison avec schéma explicatif.
- Une information sur la ceinture de grossesse.

Toutefois, les prescriptions telles que :

- Traitement antalgique médicamenteux.
- Arrêt de travail.
- Kinésithérapie…

sont laissées à la discrétion du professionnel qui suit la grossesse.

Acupuncture en complément du traitement habituel:

Les contre-indications à l'acupuncture sont les suivantes :

- Prise d'anticoagulants
- Thrombopénie connue
- Maladie hémorragique
- Trouble de l'hémostase
- Lésions cutanées

Déroulement des séances :

Le protocole de traitement nécessite un « diagnostic » au sens de la médecine traditionnelle chinoise (MTC). La durée moyenne d'une séance est d'une ½ heure à 1 heure.

a) Série initiale de 5 séances :

- 1ère semaine : 2 séances espacées au minimum de 48h
- 2ème semaine : 1 séance
- 3ème semaine : 1 séance
- 4ème semaine : 1 séance

Le délai entre les séances 2 et 3, 3 et 4 et 4 et 5 sera au minimum de 5 jours.
Si la femme enceinte se considère comme suffisamment améliorée, fin de la série initiale de 5 séances.
D'après la littérature, la plupart des essais montrant l'efficacité de l'acupuncture ont utilisés en moyenne 10 séances d'acupuncture sur 4 semaines ; soit 2 à 3 séances par semaine en moyenne.
Nous avons également sondé nos patientes sur la quantité des séances leur semblant la plus adaptée pour leur capacité de déplacement (femmes algiques) et la garantie de l'observance du traitement. D'après elles, devant le nombre important des différents rendez-vous lié au suivi de grossesse, 2 séances par semaine leur semblait difficile a honorer et présentait un frein à leur adhésion au traitement.
Lors de l'étude pilote que nous avons effectué sur 40 patientes, la fréquence des séances était de une par semaine durant 4 semaines. Les résultats une semaine après les 3 premières séances ont montrés les mêmes résultats que ceux reportés dans la littérature, soit une diminution de moitié de l'EVA.
Pour toutes ces raisons, nous avons optés pour 2 séances la première semaine et seulement une les semaines suivantes, en donnant la possibilité aux patientes de bénéficier de 5 séances supplémentaires en cas de besoin.

b) Arrêt des séances en cas d'échec

Si à l'issue de la première série, la femme enceinte ne constate aucune amélioration : fin définitive des séances d'acupuncture.

c) Séances supplémentaires en cas d'amélioration incomplète **(Schéma 0 ; Tableau 2)**

Si la femme enceinte se considère comme partiellement améliorée à l'issue de la première série de 5 séances et demande à poursuivre le traitement, une nouvelle série peut débuter sans dépasser un total de 10 séances.
Après un intervalle libre d'au moins deux semaines, une nouvelle série de séances peut être faite à la demande de la patiente, sans dépasser un total de 5 séances jusqu'à la fin de la grossesse. Le délai entre les séances sera au minimum de 5 jours. La répétions de séries de séances est interdite si la première série n'a apporté aucune modification en terme de douleur, ou si il existe une contre indication au déplacement de la patiente vers le site où est faite l'acupuncture.

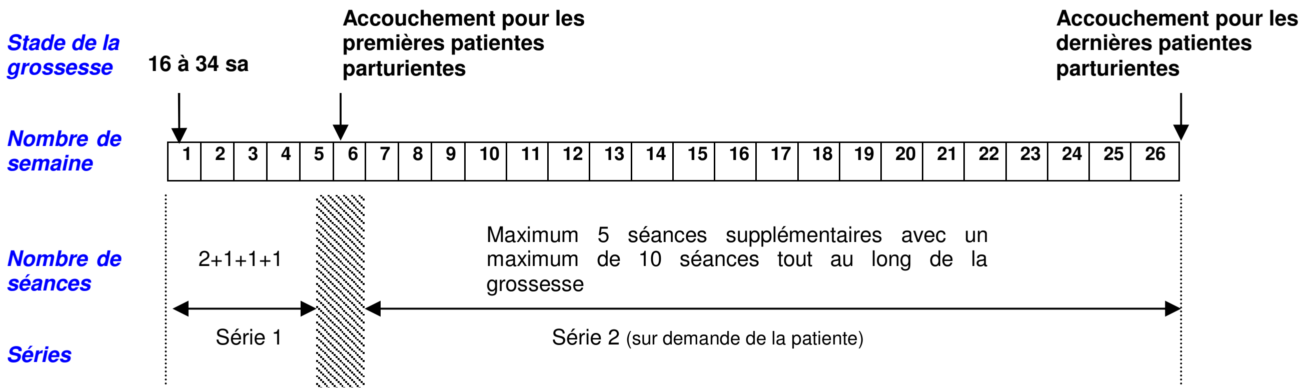

Schéma 0. Résumé de l'enchaînement des différentes séances d'acupuncture

Tableau 2. Flow Chart

Semaines		S1		S2	S3	S4	S5	S6	S7-S11	S12 à S26 maxi
**Jours**		**J1**	**J5 ±2**	**J11 ±3**	**J18 ±3**	**J25 ±3**	**J32 ±3**	**J39 ±3**	**J43 à J77**	**J78 à J182 maxi**
Screening	X									
Critères d'éligibilité	X	X								
Signature consentement éclairé et randomisation		X								
**Visites « Patientes Acupuncture »**		**V1**[a]	**V2**[a]	**V3**[a]	**V4**[a]	**V5**[a]	**V6**		**V7-V11**[b]	
Séances d'acupuncture		X	X	X	X	X			X **(option)**	
Visite bilan, EVA par la sage femme		X					X			
**Visites « Patientes Traitement Habituel »**		**V1**					**V2**			
Visite bilan, EVA par la sage femme		X					X			
Accouchement								X		
Remplissage de l'auto questionnaire pour l'ensemble des patientes (carnet de bord)		**X** *	X	X	X	X	**X** *	X		

** L'auto-questionnaire (carnet de bord) est remis aux visites V1 et V6 (ou V2 selon les patientes) mais doit être complété par la patiente tous les jours jusqu' l'accouchement. Le premier carnet de bord est remis à la V1 et récupéré à la V6 (ou V2). Le second carnet de bord est remis à la V6 (ou V2) et renvoyé par la patiente après son accouchement à l'aide d'une enveloppe prétimbrée et précomplétée.*

[a] *Le délai entre la séance d'acupuncture 1-2 est de 48h minimum. Entre les séances 2-3, 3-4 et 4-5, il sera de 5 jours minimum dans la série 1.*

[b] *Un délai de 15 jours minimum doit être respecté entre la dernière séance de la série 1 et la première séance de la série 2, le cas échéant. Le délai entre les séances dans la série 2 sera au minimum de 5 jours.*

d) Déroulement de chaque séance :

Avant et après chaque séance, sont effectués : mesure de la fréquence cardiaque, de la pression artérielle, et vérification de l'activité cardiaque fœtale.

La première séance d'acupuncture se déroule en plusieurs phases :

- Interrogatoire
- Examen des pouls
- Examen de la langue
- Etablissement d'un diagnostic en médecine traditionnelle chinoise (MTC)
  - soit de « vide de l'énergie du rein »
  - soit « stase de sang »
- Insertion des aiguilles
- Retrait des aiguilles

L'interrogatoire et l'examen clinique de la patiente vont permettre de différencier deux diagnostics:

**Diagnostics**	**Vide énergie du rein**	**Stase de sang**
**Symptômes**	Douleur diffuse, soulagée par le massage, faiblesse des genoux et des jambes, fatigue, vertiges	Douleur accentuée à la pression, fixe ou à type de piqure<br>ou accompagné de symptôme stress, anxiété
**Examen de la langue**	Pâle et grosse, ou rouge et mince	Couleur sombre, pourpre ou framboisée<br>Vaisseaux sublinguaux dilatés
**Examen des Pouls en MTC**	Vide au niveau du pied en particulier	Tendu ou normal

Déroulement de la séance :

**Position de la patiente**	Décubitus latéral, un coussin sous la tête, la jambe demie fléchie côté du décubitus, et l'autre jambe fléchie.<br><br>Nous pouvons rajouter un coussin de soutien en abdominal, pour plus de confort.
**Insertion des aiguilles**	Les aiguilles sont insérées d'une profondeur variant de 15 à 40 mm. L'acupuncteur doit rechercher la sensation du De Qi au moment de l'insertion.<br><br>Une stimulation manuelle est effectuée une seule fois, après la pose des aiguilles.
**Aiguilles utilisées**	Les aiguilles sont stériles, et à usage unique. Diamètre de 0,25 mm ou de 0,26 mm
**Durée :**	Les aiguilles sont laissées en place 30 minutes.

Protocole de points utilisés :

**Diagnostics**	**Vide énergie du rein**	**Stase de sang**
**Points utilisés quelque soit la localisation de la douleur, en fonction du diagnostic en MTC**<br>(Figure 1)	- **V23, Shenshu** : à 1,5 cun en dehors du bord inférieur de l'épineuse de la 2ème vertèbre lombaire, de chaque coté.<br><br>- **Rn9, *Zhubin* :** à 5 cuns au-dessus de Rn 3 et 1 cun en arrière du bord postérieur du tibia. L'aiguille enfoncée perpendiculairement (1 à 1,5 cun).	- **F3, Tai Chong** : entre 1er et 2ème métatarsien, à 1,5 cun du précédent, dans l'espace interosseux, à la jonction entre la base et la diaphyse du 1er métatarse.<br><br>- **Rte10, Xue Hai** : à 2 cuns au dessus de l'angle supéro-interne de la rotule.
**Quelque soit la localisation de la douleur, et quelque soit le diagnostic en MTC**<br>(Figure 1)	- **V40, Weizhong** : au milieu du creux poplité, de chaque coté.<br><br>- **Points AShi** : points locaux douloureux : l'aiguille est insérée sur les points douloureux, diagnostiqués par l'acupuncteur lors de la palpation.	
**Points supplémentaires en cas de douleurs sacroiliaques quelque soit le diagnostic en MTC**<br>(Figure 1)	**- V26, *Guanyuanshu* :** à 1,5 cun en dehors du bord inférieur de l'épineuse de la 5ème vertèbre lombaire.<br><br>**- V32, *Ci Liao*** : au niveau du 2ème trou sacré à environ 1cun en dehors de la ligne médiane.<br><br>- **Points AShi** : points locaux douloureux : l'aiguille est insérée sur les points douloureux, diagnostiqués par l'acupuncteur lors de la palpation.	
**Points supplémentaires en cas de douleurs dorsales associées, quelque soit le diagnostic en MTC**<br>(Figure 1)	Les points choisis se situent sur la branche interne des méridiens de Vessie droit et gauche au niveau de la zone douloureuse, à 1,5 cun en dehors des apophyses épineuses.<br><br>- Les aiguilles insérées tangentiellement à la peau dans le sens du méridien, trois points de chaque côté.	
**Points supplémentaires en cas de sciatalgies associées, quelque soit le diagnostic en MTC**<br>(Figure 1)	Nous rajouterons du côté atteint :<br>- **VB 30, Huantiao** : intersection 1/3 externe-2/3 interne de la ligne joignant le sommet du grand trochanter au hiatus sacro-coccygien, direction OGE.<br><br>**V 57, Chengshan** : à mi-distance entre le sillon poplité et le talon, dans un sillon en V renversé qui sépare les chefs du gastrocnémien, à 8 cuns sous V 40 (entre les deux muscles jumeaux interne et externe).	
**Points supplémentaires en cas de douleur pelvienne antérieure, quelque soit le diagnostic en MTC**<br>(Figure 1)	**- Rn11, *Hengu* :** Sur le bord supérieur du pubis et à 0,5 cun en dehors de la ligne médiane. L'aiguille enfoncée perpendiculairement (0,5 à 1 cun).<br><br>**- F6, *Zhongdu* :** A 7 cuns au-dessus de la pointe de la malléole tibiale, contre le bord médial du tibia. La piqûre, perpendiculaire ou oblique (0,3 à 0,5 cun).<br><br>**- Rn9, *Zhubin* :** A 5 cuns au-dessus de Rn 3 et 1 cun en arrière du bord postérieur du tibia. L'aiguille enfoncée perpendiculairement (1 à 1,5 cun).	

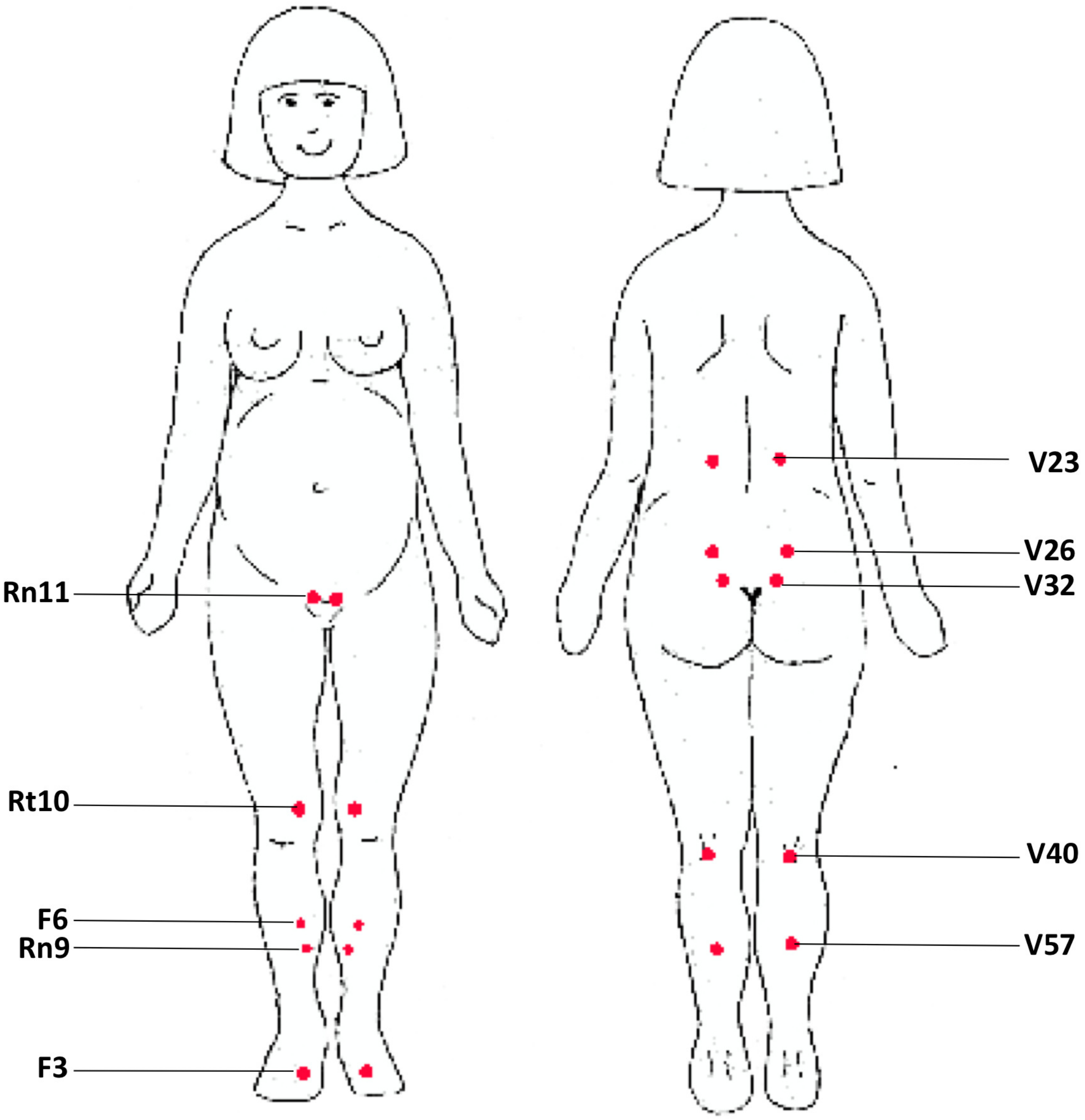

Figure 1. Schéma des points utilisés

III.2.6 Suivi après inclusion

La prise en charge obstétricale habituelle n'est par modifiée par l'inclusion dans l'essai.

La durée du suivi dans le cadre de l'essai est comprise entre l'inclusion (16 à 34 sa) et l'accouchement. Le suivi porte sur la douleur, la limitation fonctionnelle, et le recours aux soins évalués au moyen d'un carnet de bord rempli par la patiente.
Si une femme pour qui un rendez-vous était prévu ne vient pas à la séance d'acupuncture, celle-ci sera contactée par téléphone afin de connaître la raison de son absence (trop de douleurs, trop de contraintes…). La durée du traitement de la première série sera prolongée au maximum de 1 semaine.

a*) Sont notés quotidiennement par la femme enceinte sur son carnet de bord:*
- Evaluation de la douleur maximale ressentie (score de 1 à 10)
- Recours aux soins suivants (grille à cocher sur le carnet de bord + détails en texte libre) :
  - Antalgiques par voie générale
  - Consommation de produits non médicamenteux (tisanes, pommades, topiques...)
  - Consultation médicale hors urgence
  - Consultation médicale en urgence
  - Echographie
  - Arrêt de travail
  - Congé maternité supplémentaire
  - Pratiques antalgiques : kinésithérapie, ostéopathie, acupuncture, relaxation, gymnastique, massages, hypnose ou autre
  - Ceinture de grossesse
  - Usage de transports médicalisés
  - Hospitalisation (hors accouchement)

b) Evaluation hebdomadaire de l'incapacité fonctionnelle éventuelle (score d'Oswestry auto administré) notée par la femme enceinte sur son carnet de bord (tous les 7 jours par rapport au premier rendez-vous dans le bras « traitement habituel », de préférence la veille de la séance d'acupuncture dans le bras « acupuncture » puis tous les 7 jours).

Chaque semaine, la patiente est contactée par téléphone par un technicien d'étude clinique (TEC) pour l'inciter au renseignement du carnet de bord. La sage femme peut être contactée par les patientes chaque jour ouvré, par l'intermédiaire du CIC, en cas de difficulté à compléter le carnet de bord en particulier.

Après l'accouchement, le dossier obstétrical est analysé par la sage femme investigatrice et le TEC pour recueillir les données d'issue de grossesse et vérifier les données recueillies à l'inclusion (voir paragraphe III.4. Mesures prises pour éviter les biais)

III.2.7 Nombre de sujets nécessaire

On prévoit l'inclusion de 150 femmes dans chaque bras (**voir chapitre IV. Analyse statistique**)

III.3 Faisabilité

III.3.1 Compétence des investigateurs en acupuncture

Mme Nicolian, sage femme qui effectuera les séances d'acupuncture, est diplômée du DIU d'acupuncture (Paris XIII). Elle pratique régulièrement l'acupuncture, principalement dans l'indication des douleurs de la grossesse. Sa double compétence en obstétrique et en acupuncture lui permet de prendre en charge les femmes enceintes dans des conditions optimales de sécurité.

Le Dr Bingkai LIU, de nationalité chinoise, est Docteur en médecine traditionnelle chinoise (diplôme chinois). Il est également Docteur ès Sciences en France. Sa double compétence en acupuncture et en sciences fondamentales a motivé son recrutement dans le cadre du programme de médecine traditionnelle chinoise du GHPS. Le Dr Liu a contribué à la réflexion sur l'acupuncture lors de la rédaction du protocole et pourra être une personne-ressource lors du déroulement de l'étude (information sur de nouvelles publications dans le domaine de l'acupuncture).

III.3.2 Locaux dédiés et logistique

Des locaux seront dédiés au centre d'investigation clinique du Groupe Hospitalier Pitié Salpêtrière, pour effectuer les séances d'acupuncture. Le CIC assurera également la logistique (rendez vous, approvisionnement….).

III.3.3 Capacité à recruter des patientes

Une enquête par questionnaire menée auprès des femmes enceintes suivies en consultation sur le site de la Pitié nous a permis de constater que 20 femmes par mois déclarent souffrir de douleurs lombaires ou pelviennes pour lesquelles elles seraient prêtes à entrer dans un essai thérapeutique utilisant l'acupuncture. Sous l'hypothèse que seules 10% de ces femmes entreraient effectivement dans l'essai, on pourrait espérer 24 inclusions par an dans un centre.

Par ailleurs, 15 nouvelles patientes prennent actuellement chaque mois rendez vous à la consultation de Mme Nicolian, dédiée aux DLCP de la grossesse, ce qui représente 8% des nouvelles inscriptions mensuelles. Si 10 % de ces femmes acceptaient d'entrer dans un essai, le chiffre de 19 inclusions par an et par centre serait possible.

D'une façon plus globale, les centres participants prennent en charge 15 500 naissances par an. Huit pour cent de ces femmes (n=1240) présenteraient des douleurs lombaires et pelviennes suffisamment gênantes pour motiver un recours à l'acupuncture. Si 10% des femmes avec douleur gênante participaient à l'étude chaque année (n=124), 372 femmes pourraient être incluses sur 3 ans, ce qui est compatible avec notre objectif de 300 inclusions.

III.3.4 Motivation des femmes enceintes

Le traitement dit « habituel » des douleurs pelviennes de la grossesse est souvent un échec, ce qui rend attractive la possibilité d'une prise en charge par acupuncture dans le cadre d'un essai thérapeutique.

Les facteurs suivants peuvent être un frein au recrutement :

- Craintes d'une thérapeutique « exotique » (nécessité d'une information claire)
- Nécessité d'un déplacement pour les patientes incluses dans le bras « acupuncture » (inclusion et acupuncture possibles dans les centres participant à l'étude).

Les frais de transport au GHPS des patientes seront remboursés sur présentation de justificatifs à hauteur de 25 euros maximum par aller-retour. A noter que le transport médicalisé n'est pas pris en charge car si la patiente pour venir doit utiliser ce type de transport c'est qu'elle n'est pas en condition de se déplacer.

III.4 Mesures prises pour éviter les biais

La comparabilité des groupes sera vérifiée au moyen des critères suivants:

- Age maternel, taille, poids, IMC avant la grossesse à l'inclusion et à l'accouchement.
- Statut marital
- Profession
- Profession du conjoint
- Nationalité et origine géographique
- Parité et gestité
- Antécédents médicaux, chirurgicaux et obstétricaux significatifs
- Usage de tabac, alcool, autres substances
- Antécédent d'usage de l'acupuncture

III.5 Données recueillies

III.5.1 Informations recueillies à l'inclusion

- Un auto questionnaire est complété par la femme enceinte, et suivi par un interrogatoire de la sage femme.
- Mode d'adressage de la femme enceinte
- Identité de la femme enceinte (uniquement dans le dossier médical)
- Adresse et coordonnées téléphoniques ou mail (uniquement dans le dossier médical)
- Date de naissance
- Nationalité et origine géographique
- Taille poids avant la grossesse et à l'inclusion
- Mode de vie :
    - Statut marital
    - Profession de la femme enceinte
    - Patiente en activité à l'inclusion (hors arrêt de travail)
    - Port de charge lourde ou travail debout

- Temps de transport
  - Profession du conjoint
- Antécédents
  - Obstétricaux (parité, gestité...)
  - Médico-chirurgicaux (exclusion des douleurs chroniques avant la grossesse et des maladies chroniques en général)
  - Usage de tabac, alcool, autres substances
  - Antécédent d'usage de l'acupuncture
- Données de la grossesse actuelle :
  - Date de début de grossesse.
  - Site de suivi et nom du professionnel en charge du suivi (médecin ou sage femme)
  - Lieu prévu de l'accouchement
  - Absence de complication de la grossesse
- Critères d'inclusion et de non inclusion (voir paragraphe spécifique III.2.2 et III.2.3)
- Caractéristiques de la douleur :
  - Siège :
    - Ceinture pelvienne (entre les crêtes iliaques en haut et la racine des cuisses en bas, intéressant le bassin, les hanches, le pubis, les sacro iliaques, la région sacrée et fessière).
    - Rachis lombaire bas (ne dépassant pas le niveau des crêtes iliaques) ou haut
    - Rachis dorsal
  - Intensité de la douleur sur une échelle numérique (échelle de 0 à 10) et EVA (0 à 100)
- Gêne fonctionnelle (questionnaire standardisé Oswestry)

III.5.2 Informations recueillies lors du suivi

Sont notés quotidiennement par la femme enceinte sur son carnet de bord :

- Evaluation de la douleur maximale ressentie (score de 1 à 10)

Recours aux soins suivants (grille à cocher + détails en clair) :

- Consommation d'antalgiques par voie générale
- Consommation de produits non médicamenteux (tisanes, pommades, topiques..)
- Consultation médicale hors urgence
- Consultation médicale en urgence
- Echographie
- Arrêt de travail
- Congé maternité supplémentaire
- Pratiques antalgiques : kinésithérapie, ostéopathie, acupuncture, relaxation, gymnastique, massages, hypnose ou autre
- Ceinture de grossesse
- Usage de transports médicalisés
- Hospitalisation (hors accouchement)

- Evaluation hebdomadaire de l’incapacité fonctionnelle éventuelle (Score d’Oswestry) notée par la femme enceinte sur son carnet de bord

III.5.3 Protocole d’acupuncture

- Nombre de séances par femme
- Femmes ayant reçu le seul protocole de douleur lombaire basse ou de la ceinture pelvienne (N, %)
- Femmes ayant reçu en plus un protocole complémentaire pour douleurs lombaires hautes ou dorsales ou sciatalgie (N, %)
- Temps de travail représenté par les séances d’acupuncture
- Temps de déplacement et d’immobilisation des femmes lors des séances d’acupuncture

III.5.4 Perdues de vue et abandon du suivi

- Fréquence et caractéristiques

III.5.5 Rupture de protocole

- Cas de recours à l’acupuncture en dehors du protocole d’étude.
- Analyse en intention de traitée et en protocole complets.

III.5.6 Informations recueillies après l’accouchement

Ces informations sont récupérées dans le dossier obstétrical par la sage-femme ou le TEC après l’accouchement :

- Terme de naissance
- Sexe de l’enfant
- Poids de naissance
- Mode d’accouchement
    - Césarienne
    - Voie vaginale
- Score d’Apgar
- Complication maternelle survenue après l’inclusion
    - Pré éclampsie
    - Menace d’accouchement prématuré
    - Diabète gestationnel
    - Autre
    - Hospitalisation(s) en cours de grossesse (durée totale moyenne)
    - Hospitalisation lors de l’accouchement (durée moyenne)
- Complication néonatale
    - Admission en néonatologie ou en réanimation
    - Durée d’hospitalisation

III.6 Distinction soin / recherche

Ce qui relève du soin	Ce qui relève de la recherche
Le suivi de la grossesse, la prescription de traitements classiques (ceinture de grossesse, antalgiques, kinésithérapie….).<br>L'information et les conseils standardisés (postures, exercices….).	Les séances d'acupuncture.

IV. ANALYSE STATISTIQUE

L'unité d'analyse est la grossesse.
Les variables recueillies feront l'objet de statistiques descriptives.
La comparaison du groupe avec intervention (acupuncture) et du groupe témoin se fera en intention de traiter. Au cas ou des femmes randomisées sans acupuncture seraient finalement utilisatrices d'acupuncture hors protocole, ou bien ou des femmes du groupe acupuncture auraient renoncé au traitement (crainte, effet secondaire…) une analyse « per protocole » serait effectuée.

IV.1 Analyses principales

Deux analyses principales seront menées avec protection du risque de première espèce selon Bonferroni, chacune au risque 0,025. L'une des analyses sera confirmatoire, l'autre pour preuve d'effet à moyen terme de l'acupuncture tout au long de la grossesse.
La première analyse comparera la douleur maximum à 4 semaines entre les deux groupes de femmes, par un test de Mann-Whitney.
La seconde analyse reposera sur un modèle à effets mixtes exprimant, pour chaque femme et chaque observation de la douleur, la probabilité pour que, ce jour, la douleur présente un niveau inférieur ou égal à 4/10. Cette probabilité sera supposée, sur une échelle logit, somme d'un effet aléatoire patiente distribué normalement et d'un effet fixe reflétant l'apport global de l'acupuncture. La mise en évidence de l'intérêt de l'acupuncture reposera sur le test du rapport de vraisemblance. Les différentes observations réalisées chez la même femme seront supposées indépendantes conditionnellement à l'effet aléatoire.

IV.2 Analyses secondaires

Les caractéristiques cliniques et biologiques des femmes pendant la grossesse, le mode d'accouchement, les caractéristiques des nouveau-nés seront comparés entre les groupes de femmes. Ces comparaisons seront effectuées sans protection, les degrés de signification correspondants étant plutôt destinés à constater l'absence de différences.

Les distributions des scores d'incapacité fonctionnelle d'Oswestry seront comparées par le test de Mann Whitney.
On cherchera à analyser plus précisément l'effet propre de l'acupuncture, en incluant dans le modèle à effets mixtes des effets d'interaction, notamment avec le terme et le délai depuis la dernière séance d'acupuncture (recherche de la persistance de l'effet), et le terme comme covariable d'ajustement.
Les analyses seront effectuées à l'aide du logiciel SAS Version 9.2 (SAS, Cary Road, NY).

IV.3 Nombre de sujets nécessaires

L'étude est dimensionnée sur la base de l'objectif principal qui s'administre en deux temps et à travers deux analyses statistiques : 1) confirmation par un test de comparaison de distributions de l'effet de l'acupuncture sur la douleur à 4 semaines; 2) mise en évidence de l'effet global de l'acupuncture sur la douleur durant toute le grossesse, par comparaison des couples (nombre de jours sans douleur supérieure à 4/10, nombre de jours avec douleur supérieure à 4/10) entre les deux groupes de femmes. Le succès de l'étude étant acquis si l'une de ces deux analyses est positive, chacun des tests correspondants sera effectué au seuil de significativité de 0,025. Deux calculs de nombre de sujets nécessaires sont réalisés.

1. On suppose, en se fondant sur les données de Elden (Elden, 2005), que quatre semaines après l'inclusion, les femmes du groupe témoin présenteront une douleur maximum moyenne de 5/10, les femmes du groupe acupuncture une douleur maximum moyenne de 3/10. En exploitant les inter quartiles de l'article de Elden, on peut estimer l'écart type de la douleur à l'inclusion et à 4 semaines à 0,21 environ. Si l'on suppose que la corrélation entre l'évolution de la douleur (différence douleur à l'inclusion et douleur à 4 semaines) et le niveau initial de douleur est de 0,7 (le niveau de douleur expliquant alors la moitié de la variabilité de l'évolution), on peut estimer à 0,3 l'écart type de l'évolution du niveau de douleur. Avec de telles valeurs, et pour assurer au test de comparaison (Mann et Whitney) une puissance de 80%, il faut inclure 49 femmes par groupe.
2. Le nombre de sujets nécessaires pour la seconde analyse de l'objectif principal repose sur une simulation reproduisant les données conformément aux hypothèses et modèle présentés ci-dessus, auxquels on adjoint la dimension aléatoire suivante : on suppose que la douleur un jour donné est égale à la douleur de la veille à laquelle s'ajoute une perturbation aléatoire gaussienne d'écart type 0,2/70 de telle sorte que sur la durée moyenne de participation d'une femme à l'étude (70 jours) les trajectoires de deux femmes présentant à 4 semaines le même niveau de douleur puissent différer de l'ordre de 0,4. Il s'agit donc d'un modèle conférant aux trajectoires de douleur un assez haut niveau de variabilité inter jour. Par ailleurs on suppose que les femmes du groupe témoin présentent pendant toute la grossesse un niveau moyen de douleur de 5/10, les femmes du groupe acupuncture une évolution sur 70 jours de 3/10 à 4/10, matérialisée par l'espérance de la perturbation précédente, La simulation montre que la proportion moyenne de jours avec douleur inférieure à 4/10 serait de 52% environ chez les femmes du groupe acupuncture, et de 30% dans le groupe de femmes témoins. Les logits de la probabilité pour qu'un jour soit un jour de douleur inférieure à 4/10 peuvent être considérés comme distribués

normalement avec respectivement des moyennes de –1,1 et 0,3, pour une valeur commune de l'écart type de 3,7. La véritable analyse reposera, on l'a dit, sur un modèle à effet aléatoire reflétant fidèlement la structure des données, et notamment leur recueil récurrent, sur des durées variables. Le calcul de planification qui suit est plus simple, et exploite la distribution supposée des logits, sans tenir compte de la variabilité de la durée de suivi des femmes, le test résultant étant une simple comparaison de moyennes ; pour assurer à ce test une puissance de 80%, il faut inclure 135 femmes dans chacun des groupes, nombre permettant d'assurer une haute puissance à la première analyse. Nous proposons finalement d'inclure 150 femmes par groupe pour tenir compte de la variabilité des durées de suivi.

IV.4 Analyse économique

L'étude économique porte sur :

Le coût total de chaque stratégie :

- du point de vue du système de soins
- du point de vue de l'Assurance Maladie
- du point de vue des patientes,

Quoique les dépenses liées à la grossesse soient prises en charges à 100% par l'Assurance Maladie, les dépassements d'honoraires pour certaines consultations ou les recours aux médecines complémentaires (en dehors du protocole) constituent un reste à charge. De même, la différence entre les Indemnités Journalières et le salaire réel représente un manque à gagner pour les femmes.

L'horizon temporel est la durée de la grossesse.

Le **tableau 2** récapitule les données recueillies, le mode de recueil, le mode de valorisation et le point de vue pertinent pour chaque ressource. On peut restreindre le périmètre du système de soins au seul hôpital et dans ce cas les seules ressources pertinentes sont celles décrites ci-dessous au 1). Les autres ressources du système de soins sont les consommations ambulatoires de biens et soins médicaux. Les ressources pertinentes du point de vue de l'Assurance Maladie sont celles qui sont inscrites dans une nomenclature nationale. Les ressources pertinentes du point de vue des patientes sont celles qui font l'objet d'un reste à charge.

Tableau 2. Résumé des données recueillies

Ressource	Mode de recueil	Valorisation
1) Temps passé par les professionnels hospitaliers pour la prise en charge des douleurs :<br>o Obstétricien<br>o Sage femmes<br>o Acupuncteur	Cahier d'observation<br>Ce recueil concerne les consommations prévues au protocole et celles non prévues au protocole	Salaire chargé AP-HP<br><br>Facturation hospitalière à l'Assurance Maladie
2) Recours à des professionnels non hospitaliers motivé par la douleur :<br>o Médecin libéral<br>o Kinésithérapeute<br>o Autre acupuncteur<br>o Ostéopathe<br>o Ergothérapeute<br>…	Carnet de bord remis à la patiente	Remboursement par l'Assurance Maladie selon les nomenclatures nationales<br>ET<br>Reste à charge pour la patiente
3) Transports médicalisés	Carnet de bord remis à la patiente	Remboursement par l'Assurance Maladie<br>ET<br>Reste à charge pour la patiente
4) Traitements prescrits	Cahier d'observation	Remboursement par l'Assurance Maladie
5) Autres traitements non prescrits (médecines complémentaires ou alternatives)	Carnet de bord remis à la patiente	Dépense de la patiente
6) Arrêts maladie prescrits	Cahier d'observation	Indemnités journalières ET manque à gagner pour la patiente

Le recueil des informations nécessaires pour l'évaluation économique implique la réalisation d'un carnet de bord, préformaté remis à chaque patientes et sur lequel les patientes notent leurs consommations entre 2 visites de suivi. Afin de minimiser le risque de perte, le premier carnet de bord sera remis par la sage-femme à la visite d'inclusion/randomisation lors de la V1 pour la période V1-V6. Puis à la V6, le premier carnet de bord est récupéré et un second carnet de bord est remis à la patiente, pour la période V6 jusqu'à l'accouchement. Celui-ci devra être renvoyé par la patiente après l'accouchement dans une enveloppe pré-timbrée et précomplétée qui aura été fournie à la V6. Les autres informations seront recueillies sur le dossier patient au moment de chaque visite.

L'utilisation d'un carnet de bord pour recueillir des informations sur les consommations de soins expose à plusieurs biais, mais c'est le seul mode de recueil possible compte tenu du fait 1) que les données de remboursement de l'assurance Maladie ne seront pas disponibles et 2) que des consommations de ressources qui nous intéressent sont hors du champ du remboursement. On peut imaginer plusieurs types de biais et nous n'avons actuellement pas d'hypothèse sur les plus vraisemblables : que les femmes du groupe contrôle soient moins motivées et remplissent mal le carnet de bord ce qui surestimerait par comparaison les consommations du groupe acupuncture ; que les femmes du groupe acupuncture ne rapportent pas leurs autres consommations de médecines complémentaires ou de médicaments ; que les professionnels soient plus réticents à prescrire des antalgiques dans le groupe acupuncture et qu'il y ait de l'automédication…En l'absence de données de la littérature sur lesquelles fonder une stratégie de recueil d'information, la rigueur du recueil reposera principalement sur les TECs qui seront sensibilisés à la particulière importance des entretiens téléphoniques de suivi.

Nous faisons l'hypothèse que la prise en charge de la douleur ne nécessitera pas d'hospitalisation, quel que soit le bras de traitement. Toutefois, les hospitalisations en cours d'étude constituent des événements indésirables graves et sont recueillies systématiquement. S'il s'avérait que certaines hospitalisations sont imputables à la douleur, celles-ci seraient comptabilisées et feraient l'objet d'une valorisation dans le cadre de l'évaluation économique.

Comparaison des ressources consommées :
L'analyse des coûts du point de vue du système de soins, de l'Assurance Maladie et des femmes sera effectuée en intention de traiter. Outre les statistiques descriptives, nous comparerons les consommations et les coûts en identifiant d'abord le type de distribution (recherche du meilleur fit, le plus souvent les coûts sont distribués selon une fonction bêta, mais on teste également la fonction log normale).
Dans l'hypothèse ou les surcoûts engendrés par les séances d'acupuncture sont compensés par une réduction de consommations d'antalgiques, de consultations et éventuellement d'arrêts maladie, ET si la qualité de vie dans le groupe acupuncture est meilleure, nous serons dans la situation simple de stratégie dominante.
Si les surcoûts de l'acupuncture ne sont que partiellement compensés par les réductions de consommation, mais toujours avec une meilleure qualité de vie, nous estimerons un surcoût par journée de douleur évitée. En d'autres termes, nous estimerons la différence de coût entre les deux bras, divisée par la différence du nombre de jours où la douleur est supérieure au seuil de 4/10.

Analyse de sensibilité :
Il sera nécessaire de réaliser plusieurs types d'analyse de sensibilité. D'une part des analyses uni variées en testant différentes hypothèses sur les professionnels impliqués dans l'acupuncture et en faisant varier les salaires. D'autre part des analyses par bootstrap sur les consommations de ressources et sur le bénéfice clinique. Pour cela il sera nécessaire de déterminer aussi le modèle distribution des bénéfices cliniques (nombre de jours sans antalgiques ou nombre de jours avec une douleur inférieure ou égale à 4/10).

V. RESULTATS ATTENDUS ET PERSPECTIVES.

V.1 Résultats attendus

Femmes perdues de vues et abandon du suivi : 10%
Rupture de protocole : 10%

V.2 Critère de jugement principal

Dans le groupe acupuncture + traitement habituel, on attend :

- une évaluation de la douleur moyenne après 4 semaines proche de 3/10 contre une évaluation de la douleur moyenne en cours de grossesse proche de 5 /10 dans le groupe avec traitement habituel seul.

- un % de journées avec douleur maximale > 4/10 de l'ordre de 50 % dans le groupe acupuncture et de l'ordre de 70% dans le groupe témoin.

V.3 Critères secondaires

Dans le groupe acupuncture + traitement habituel, on attend, en comparaison du groupe avec traitement habituel seul:

- Une réduction de 20 % de l'indice d'invalidité moyen mesuré entre l'inclusion et l'accouchement.
- Une diminution de 20% de la consommation d'antalgiques
- Pas de modification des indicateurs de morbidité obstétricale et périnatale

Le coût global des soins de santé et des conséquences de l'invalidité, mesuré entre l'inclusion et l'accouchement, sera évalué dans les deux groupes.

V.4 Perspectives

Nos résultats pourraient contribuer à éclairer les institutions hospitalières sur le choix d'offrir ou non l'acupuncture comme traitement complémentaire en obstétrique.

VI. TRAITEMENT ADMINISTRE AUX PERSONNES QUI SE PRETENT A LA RECHERCHE

En application des obligations des Bonnes Pratiques de fabrication en date du 26 mai 2006, une carte patient sera remise systématiquement portant la mention « Merci de garder cette carte en permanence avec vous » et devant préciser le nom, l'adresse et le n° de téléphone de l'investigateur, voire du contact principal (si différent) pour les informations sur le produit expérimental ou la recherche, le code de la recherche, le n° de la patiente.

VII. EVALUATION DE LA SECURITE

Aucun effet indésirable de l'acupuncture sur le déroulement de la grossesse n'a été décrit.

Tout évènement imprévu sera signalé au CIC par la patiente elle-même ou par les professionnels de santé concernés (contact téléphone, mail, et fax figurant sur la carte de la patiente).

VII.1 Description des paramètres d'évaluation de la sécurité

- **Evènement indésirable**
  Toute manifestation nocive survenant chez une personne qui se prête à une recherche biomédicale, que cette manifestation soit liée ou non à la recherche ou au produit sur lequel porte cette recherche (Art. R. 1123-39 – Décret n°2006-477 du 26 avril 2006).

- **Effet indésirable d'un dispositif médical**
  Toute réaction nocive et non désirée à un dispositif médical ou tout incident qui aurait pu entraîner cette réaction si une action appropriée n'avait pas été effectuée, chez une personne qui se prête à la recherche ou chez l'utilisateur du dispositif médical ou tout effet lié à une défaillance ou une altération d'un dispositif médical de diagnostic *in vitro* et néfaste pour la santé d'une personne qui se prête à la recherche » (Art. R. 1123-39 – Décret n°2006-477 du 26 avril 2006).

- **Evènement ou effet indésirable grave**
  Tout évènement ou effet indésirable qui entraîne la mort, met en danger la vie de la personne qui se prête à la recherche, nécessite une hospitalisation ou la prolongation de l'hospitalisation, provoque une incapacité ou un handicap importants ou durables, ou bien se traduit par une anomalie ou une malformation congénitale.

- **Effet indésirable inattendu d'un dispositif médical**
  Tout effet indésirable du dispositif médical ou du dispositif médical de diagnostic in vitro dont la nature, la sévérité ou l'évolution ne concorde pas avec les informations figurant respectivement dans la notice d'instruction ou la notice d'utilisation lorsqu'il fait l'objet d'un marquage CE, et dans le protocole ou la brochure pour l'investigateur lorsqu'il ne fait pas l'objet d'un tel marquage. (Art-R1221-23 – Décret n°2006-477 du 26 avril 2006).

- **Fait nouveau**
  Toute nouvelle donnée de sécurité, pouvant conduire à une réévaluation du rapport des bénéfices et des risques de la recherche ou du dispositif médical, ou qui pourrait être suffisant pour envisager des modifications dans l'utilisation du dispositif médical, dans la conduite de la recherche.

VII.2 Méthodes et calendrier prévus pour mesurer, recueillir et analyser les paramètres d'évaluation de la sécurité

VII.2.1 Comité de pilotage

Il sera constitué des initiateurs cliniciens du projet, du biostatisticien en charge du projet, des représentants du promoteur et de l'URC nommés pour cette recherche.

Il définira l'organisation générale et le déroulement de la recherche et coordonnera les informations.
Il déterminera initialement la méthodologie et décidera en cours de recherche des conduites à tenir dans

les cas imprévus, surveillera le déroulement de la recherche en particulier sur le plan de la tolérance et des évènements indésirables.

VII.2.2 Comité de surveillance indépendant

Il n'est pas décidé de créer un tel comité car la recherche ne présente pas de risque.

VII.3 Procédures mises en place en vue de l'enregistrement et de la notification des évènements indésirables

VII.3.1 Evènements indésirables non graves

Tout événement indésirable - non grave suivant la définition précédente - observé lors de la recherche et dans ses suites devra être reporté dans le cahier d'observation dans la section prévue à cet effet.

Un seul évènement doit être reporté par item. L'évènement peut correspondre à un symptôme, un diagnostic ou à un résultat d'examen complémentaire jugé significatif. Tous les éléments cliniques ou para-cliniques permettant de décrire au mieux l'évènement correspondant doivent être reportés.

VII.3.2 Evènements indésirables graves (EIG)

Le formulaire de déclaration d'un événement indésirable grave, validé pour la recherche, est inclus dans le protocole en annexe. Il en est de même pour la grille de classification des événements indésirables graves et non graves. Cette grille a été mise en place afin d'aider l'investigateur dans sa gestion des événements indésirables (c'est-à-dire l'aider à différencier les événements selon leur gravité). La grille est élaborée et validée par l'ensemble des acteurs impliqués dans la recherche (à savoir : le Responsable de l'Unité de Recherche Clinique, l'Investigateur Coordonnateur de la recherche, le Chef de Projet de l'étude, le Coordonnateur médical du DRCD et le Responsable de la Pharmacovigilance du DRCD). Elle peut être amenée à évoluer, au décours de la recherche, en fonction des déclarations reçues par le promoteur.

Les investigateurs doivent notifier **immédiatement** au promoteur AP-HP les évènements indésirables graves tels que définis ci-dessus.

L'investigateur complète les formulaires d'évènements indésirables graves (du cahier d'observation de la recherche) et les envoie au DRCD par fax au 01 44 84 17 99 et ce, dans les 48 heures (après si possible un appel téléphonique immédiat au 01 44 84 17 23 en cas de décès ou d'une menace vitale).

L'investigateur doit également informer l'URC en charge de la recherche de la survenue de l'EIG.

Pour chaque évènement indésirable grave, **l'investigateur devra émettre un avis sur le lien de causalité de l'évènement avec chaque dispositif médical et les autres traitements éventuels.**

L'obtention d'informations relatives à la description et l'évaluation d'un évènement indésirable peuvent

ne pas être possibles dans le temps imparti pour la déclaration initiale.

Aussi, l'évolution clinique ainsi que les résultats des éventuels bilans cliniques et des examens diagnostiques et/ou de laboratoire, ou toute autre information permettant une analyse adéquate du lien de causalité seront rapportés :

- soit sur la déclaration initiale d'EIG s'ils sont immédiatement disponibles,
- soit ultérieurement et le plus rapidement possible, en envoyant par fax une nouvelle déclaration d'EIG complétée (et en précisant qu'il s'agit d'un suivi d'EIG déclaré et le numéro de suivi).

Toutes les déclarations faites par les investigateurs devront identifier chaque sujet participant à la recherche par un numéro de code unique attribué à chacun d'entre eux.

En cas de décès notifié d'un sujet participant à la recherche, l'investigateur communiquera au promoteur tous les renseignements complémentaires demandés (compte-rendu d'hospitalisation, résultats d'autopsie…).

Tout fait nouveau survenu dans la recherche ou dans le contexte de la recherche, provenant de données de la littérature ou de recherches en cours, devra être notifié au promoteur.

VII.3.3 Déclaration des évènements indésirables graves aux Autorités de Santé

Elle sera assurée par le Pôle de Pharmacovigilance du DRCD, après évaluation de la gravité de l'évènement indésirable, du lien de causalité avec chaque dispositif médical de l'étude et les autres traitements éventuels ainsi que du caractère inattendu des effets indésirables.
Toutes les suspicions d'effet indésirable grave inattendu seront déclarées par le promoteur aux autorités compétentes dans les délais légaux.

Toute donnée de sécurité ou tout fait nouveau qui pourrait modifier significativement l'évaluation du rapport des bénéfices et des risques d'un dispositif médical utilisé dans la recherche, ou de la recherche, ou qui pourrait conduire à envisager des modifications concernant les modalités d'utilisation du dispositif médical ou la conduite de la recherche, sera transmise par le promoteur aux autorités compétentes, au Comité de Protection des Personnes et aux investigateurs de la recherche.

Par exemple :

a) toute augmentation cliniquement significative de la fréquence d'apparition d'un effet indésirable grave attendu ;
b) des suspicions d'effet indésirable grave inattendu survenus chez des participants ayant terminé la recherche et qui sont notifiés par l'investigateur au promoteur, ainsi que des rapports de suivi éventuels ;
c) tout fait nouveau concernant le déroulement de la recherche ou le développement du dispositif

médical, lorsque ce fait nouveau est susceptible de porter atteinte à la sécurité des participants. A titre d'exemple :

- un événement indésirable grave susceptible d'être lié aux investigations et aux procédures de diagnostic de la recherche et qui pourrait modifier le déroulement de cette recherche,
- un risque significatif pour la population de la recherche comme par exemple un manque d'efficacité du dispositif médical utilisé dans le traitement d'une maladie mettant en jeu le pronostic vital,
- des résultats significatifs de sécurité issus d'une étude menée chez l'animal récemment terminée
- un arrêt anticipé ou une interruption temporaire pour des raisons de sécurité d'une recherche conduit avec le même dispositif médical dans un autre pays,
- un effet indésirable grave inattendu lié à un médicament non expérimental nécessaire à la réalisation de l'essai (ex : « challenge agents », traitement de secours)

d) les recommandations du comité de surveillance indépendant [Data Monitoring Committee (DMC) ou Data Safety Monitoring Board (DSMB)], le cas échéant, si elles sont pertinentes pour la sécurité des personnes,

e) tout effet indésirable grave inattendu transmis au promoteur par un autre promoteur d'une recherche biomédicale mené dans un pays tiers portant sur le dispositif médical.

VII.4 Dispositions à prendre en vue d'assurer la sécurité en cas de défaillance du dispositif médical y compris en cas de dysfonctionnement isolé du dispositif sans retentissement clinique ainsi qu'en cas de mauvaise utilisation

Toute patiente présentant un évènement indésirable doit être suivi jusqu'à la résolution ou la stabilisation de celui-ci.

- Si l'évènement n'est pas grave, l'évolution en sera notée sur la page correspondante du cahier d'observation à la section prévue à cet effet.
- Si l'évènement est grave, un suivi d'EIG sera envoyé au DRCD.

VII.5 Le protocole décrit les risques possibles et les effets indésirables prévisibles sur la base des données disponibles

Il n'a pas été mis en évidence à ce jour d'effet indésirable de l'acupuncture sur la grossesse. Toutefois, des symptômes mineurs au niveau des points de ponction sont possibles.

VIII. DROIT D'ACCES AUX DONNEES ET DOCUMENTS SOURCE

Les personnes ayant un accès direct conformément aux dispositions législatives et réglementaires en vigueur, notamment les articles L.1121-3 et R.5121-13 du code de la santé publique (par exemple, les investigateurs, les personnes chargées du contrôle de qualité, les moniteurs, les assistants de recherche clinique, les auditeurs et toutes personnes appelées à collaborer aux

essais) prennent toutes les précautions nécessaires en vue d'assurer la confidentialité des informations relatives aux médicaments expérimentaux, aux essais, aux personnes qui s'y prêtent et notamment en ce qui concerne leur identité ainsi qu'aux résultats obtenus. Les données collectées par ces personnes au cours des contrôles de qualité ou des audits sont alors rendues anonymes.

IX. CONTROLE ET ASSURANCE DE LA QUALITE

La recherche sera encadrée selon les procédures opératoires standard du promoteur.

Le déroulement de la recherche dans les centres investigateurs et la prise en charge des sujets seront réalisés conformément à la réglementation en vigueur.

IX.1 Procédures de monitoring

Le niveau de risque de la recherche est le **risque B**.

Les ARCs représentants du promoteur effectueront des visites des centres investigateurs au rythme correspondant au schéma de suivi des patients dans le protocole, aux inclusions dans les différents centres et au niveau de risque qui a été attribué à la recherche.

- **Visite d'ouverture de chaque centre :** avant inclusion, pour une mise en place du protocole et prise de connaissance avec les différents intervenants de la recherche biomédicale.

- **Lors des visites suivantes**, les cahiers d'observation seront revus au fur et à mesure de l'état d'avancement de la recherche par les ARC. L'investigateur principal de chaque centre ainsi que les autres investigateurs qui incluent ou assurent le suivi des personnes participant à la recherche s'engagent à recevoir les ARC à intervalles réguliers.
Lors de ces visites sur site et en accord avec les Bonnes Pratiques Cliniques, les éléments suivants seront revus :

- Respect du protocole et des procédures définies pour la recherche,
- Vérification des consentements éclairés des patients
- Examen des documents source et confrontation avec les données reportées dans le cahier d'observation quant à l'exactitude, les données manquantes, la cohérence des données selon les règles édictées par les procédures du DRCD.

- **Visite de fermeture** : récupération des cahiers d'observation, bilan à la pharmacie, documents de la recherche biomédicale, archivage.

IX.2 Transcription des données dans le cahier d'observation

Toutes les informations requises par le protocole doivent être fournies dans le CRF et une explication donnée par l'investigateur pour chaque donnée manquante.
Les données devront être transférées dans les CRF au fur et à mesure qu'elles sont obtenues qu'il

s'agisse de données cliniques ou para-cliniques.

Les données devront être copiées de façon nette et lisible à l'encre noire dans les cahiers (ceci afin de faciliter la duplication et la saisie informatique).

Les données erronées dépistées sur les CRF seront clairement barrées et les nouvelles données seront copiées sur le cahier avec les initiales et la date par le membre de l'équipe de l'investigateur qui aura fait la correction.

L'anonymat des sujets sera assuré par un numéro de code et les initiales de la personne qui se prête à la recherche sur tous les documents nécessaires à la recherche (3 digits pour le centre / 4 digits pour le numéro de patient / 2 digits pour les initiales : └┴┴┘ └┴┴┴┘ └┴┘), ou par effacement par les moyens appropriés des données nominatives sur les copies des documents source, destinés à la documentation de la recherche.

Les données informatisées sur un fichier seront déclarées à la CNIL selon la procédure adaptée au cas.

X. CONSIDERATIONS LEGALES ET ETHIQUES

Le promoteur est défini par la loi 2004-806 du 9 août 2004. Dans cette recherche, l'AP-HP est le promoteur et le Département de la Recherche Clinique et du Développement (DRCD) en assure les missions réglementaires.

Avant de démarrer la recherche, chaque investigateur fournira au représentant du promoteur de la recherche une copie de son **curriculum vitæ personnel daté et signé** et comportant son numéro *RPPS*

X.1 Demande d’autorisation auprès de l’ANSM

Pour pouvoir démarrer la recherche, l’AP-HP en tant que promoteur doit soumettre un dossier de demande d’autorisation auprès de l'autorité compétente l’ANSM. L'autorité compétente, définie à l'article L. 1123-12, se prononce au regard de la sécurité des personnes qui se prêtent à une recherche biomédicale, en considérant notamment la sécurité et la qualité des produits utilisés au cours de la recherche conformément, le cas échéant, aux référentiels en vigueur, leur condition d'utilisation et la sécurité des personnes au regard des actes pratiqués et des méthodes utilisées ainsi que les modalités prévues pour le suivi des personnes.

X.2 Demande d’avis au Comité de Protection des Personnes

En accord avec l'article L.1123-6 du Code de Santé Publique, le protocole de recherche doit être soumis par le promoteur à un Comité de Protection des Personnes L'avis de ce comité est notifié à l’autorité compétente par le promoteur avant le démarrage de la recherche.

X.3 Modifications

Le DRCD doit être informé de tout projet de modification du protocole par l'investigateur coordonnateur.
Les modifications devront être qualifiées en substantielles ou non.

Une modification substantielle est une modification susceptible, d'une manière ou d'une autre, de modifier les garanties apportées aux personnes qui se prêtent à la recherche biomédicale (modification d'un critère d'inclusion, prolongation d'une durée d'inclusion, participation de nouveaux centres,…).

Après le commencement de la recherche, toute modification substantielle de celle-ci à l'initiative du promoteur doit obtenir, préalablement à sa mise en œuvre, un avis favorable du comité et une autorisation de l'autorité compétente. Dans ce cas, si cela est nécessaire, le comité s'assure qu'un nouveau consentement éclairé des personnes participant à la recherche est bien recueilli.

Par ailleurs, toute extension de la recherche (modification profonde du schéma thérapeutique ou des populations incluses, prolongation des traitements et ou des actes thérapeutiques non prévus initialement dans le protocole) devra être considérée comme une nouvelle recherche.
Toute modification substantielle devra faire l'objet **<u>par le promoteur</u>** d'une demande d'autorisation auprès de l'ANSM et/ou d'une demande d'avis du CPP.

X.4 Déclaration CNIL

La loi prévoit que la déclaration du fichier informatisé des données personnelles collectées pour la recherche doit être faite avant le début effectif de la recherche.
Une méthodologie de référence spécifique au traitement de données personnelles opéré dans le cadre des recherches biomédicales définies par la loi 2004-806 du 9 août 2004 car entrant dans le champ des articles L.1121-1 et suivants du Code de Santé Publique a été établie par la CNIL en janvier 2006.

Cette méthodologie permet une procédure de déclaration simplifiée lorsque la nature des données recueillies dans la recherche est compatible avec la liste prévue par la CNIL dans son document de référence.
Lorsque le protocole bénéficie d'un contrôle qualité des données par un ARC représentant le promoteur et qu'il entre dans le champ d'application de la procédure simplifiée CNIL, le DRCD en qualité de promoteur demandera au responsable du fichier informatique de s'engager par écrit sur le respect de la méthodologie de référence MR001 simplifiée.

X.5 Note d'information et Consentement éclairé

Le consentement éclairé écrit de toutes les personnes qui se prêtent à la recherche sera obtenu par le médecin ou la sage femme déclaré en tant qu'investigateur auprès du promoteur avant que tout acte soit pratiqué dans le cadre du protocole de la recherche et quel que soit cet acte, conformément à la réglementation.

L'investigateur s'engage à obtenir le consentement éclairé de toutes les patientes avant leur inclusion. La patiente aura la possibilité de poser toutes les questions qu'elle souhaite et disposer d'un temps de réflexion avant de signer le consentement de participation. Ce consentement éclairé sera daté et signé en trois exemplaires par la patiente et l'investigateur. Une copie de ce document sera remise à la patiente, l'original sera conservé par l'investigateur dans ses archives pendant un minimum de 15 ans, une autre copie sera remise à la fin de l'essai au promoteur sous enveloppe scellée.

X.6 Rapport final de la recherche

Le rapport final de la recherche sera écrit en collaboration par le coordonnateur et le biostatisticien pour cette recherche. Ce rapport sera soumis à chacun des investigateurs pour avis. Une fois qu'un consensus aura été obtenu, la version finale devra être avalisée par la signature de chacun des investigateurs et adressée au promoteur dans les meilleurs délais après la fin effective de la recherche. Un rapport rédigé selon le plan de référence de l'autorité compétente doit être transmis à l'autorité compétente ainsi qu'au CPP dans un délai de un an, après la fin de la recherche, s'entendant comme la dernière visite de suivi du dernier sujet inclus. Ce délai est rapporté à 90 jours en cas d'arrêt prématuré de la recherche.

XI. TRAITEMENT DES DONNEES ET CONSERVATION DES DOCUMENTS ET DES DONNEES RELATIVES A LA RECHERCHE

Les documents d'une recherche entrant dans le cadre de la loi sur les recherches biomédicales doivent être archivés par toutes les parties pendant une durée de 15 ans après la fin de la recherche.

Cet archivage indexé comporte :

- Les copies de courrier d'autorisation de l'ANSM et de l'avis obligatoire du CPP
- Les versions successives du protocole (identifiées par le n° de version et la date de version),
- Les courriers de correspondance avec le promoteur,
- Les consentements signés des sujets sous pli cacheté (dans le cas de sujets mineurs signés

par les titulaires de l'autorité parentale) avec la liste ou registre d'inclusion en correspondance,

- Le cahier d'observation complété et validé de chaque sujet inclus,
- Toutes les annexes spécifiques à l'étude,
- Le rapport final de l'étude provenant de l'analyse statistique et du contrôle qualité de l'étude (double transmis au promoteur).
- Les certificats d'audit éventuels réalisés au cours de la recherche

La base de données ayant donné lieu à l'analyse statistique doit aussi faire l'objet d'archivage par le responsable de l'analyse (support papier ou informatique).

XII. ASSURANCE ET ENGAGEMENT SCIENTIFIQUE

XII.1 Assurance

L'Assistance Publique-Hôpitaux de Paris est le promoteur de cette recherche. En accord avec la loi sur les recherches biomédicales, elle a pris une assurance auprès de la compagnie HDI GERLING pour toute la durée de la recherche, garantissant sa propre responsabilité civile ainsi que celle de tout intervenant (médecin ou personnel impliqué dans la réalisation de la recherche) (loi n°2004-806, Art L.1121-10 du CSP).
L'Assistance Publique - Hôpitaux de Paris se réserve le droit d'interrompre la recherche à tout moment pour des raisons médicales ou administratives; dans cette éventualité, une notification sera fournie à l'investigateur.

XII.2 Engagement de responsabilités

Chaque investigateur s'engagera à respecter les obligations de la loi et à mener la recherche selon les B.P.C., en respectant les termes de la déclaration d'Helsinki en vigueur. Pour ce faire, un exemplaire de l'engagement des responsabilités (document type DRCD) daté et signé par chaque investigateur de chaque service clinique d'un centre participant sera remis au représentant du promoteur.

XIII. REGLES RELATIVES A LA PUBLICATION

L'AP-HP est propriétaire des données et aucune utilisation ou transmission à un tiers ne peut être effectuée sans son accord préalable.
Seront premiers signataires des publications, les personnes ayant réellement participé à l'élaboration du protocole et son déroulement ainsi qu'à la rédaction des résultats.

Par précaution, un comité d'écriture devrait être constitué et l'ordre des signataires pourra être défini par avance.

L'Assistance Publique-Hôpitaux de Paris doit être mentionnée comme étant le promoteur de la recherche biomédicale et comme soutien financier le cas échéant. Les termes « Assistance Publique-Hôpitaux de Paris » doivent apparaître dans l'adresse des auteurs.

XIV. BIBLIOGRAPHIE

1. **Albert H, Godskesen M,Westergaard J.** Evaluation of clinical tests used in classification procedures in pregnancy-related pelvic joint pain. Eur Spine J 2000;9:161-6

2. **Elden H, Fagevik-Olsen M, Ostgaard HC, Stener-Victorin E, Hagberg H.** Acupuncture as an adjunct to standard treatment for pelvic girdle pain in pregnant women: randomised double-blinded controlled trial comparing acupuncture with non-penetrating sham acupuncture. BJOG. 2008 Dec;115(13):1655-68. Epub 2008 Oct 15

3. **Elden H, Ladfors, Fagevik Olsen M, Ostgaard HC, Hagberg H**. Effects of acupuncture and stabilising exercises as adjunct to standard treatment in pregnant women with pelvic girdle pain: randomised single blind controlled trial BMJ, doi:10.1136/bmj.38397.507014.E0 (published 18 March 2005)

4. **Guerreiro da Silva JB, Nakamura MU, Cordeiro JA,Kulay L Jr.** Acupuncture for low back pain in pregnancy—a prospective, quasi-randomised, controlled study. Acupunct Med. 2004;22:60–67.

5. **Gutke A et al.** Impact of postpartum lumbopelvic pain on disability, pain intensity, health-related quality of life, activity level, kinesiophobia, and depressive symptoms. Eur Spine J. EPUB 1/7/2010

6. **Gutke A, Ostgaard HC, Oberg B.** Pelvic girdle pain and lumbar pain in pregnancy: a cohort study of the consequences in terms of health and functioning. Spine. 2006;31:E149–E155.

7. **Hansen A, Jensen DV,Wormslev M, et al.** Symptom giving pelvic girdle relaxation in pregnancy. II: symptoms and clinical signs. Acta Obstet Gynecol Scand. 1999;78:111–115.32

8. **Kvorning N, Holmberg C, Grennert L, Aberg A, and Akeson J**. Acupuncture relieves pelvic and low-back pain in late pregnancy Acta Obstet Gynecol Scand 2004: 83: 246—250

9. **Lile J, Perkins J, Hammer RL, Loubert PV.** Diagnostic and management strategies for pregnant women with back pain. JAAPA. 2003;16:31–44.

10. **Lund I, Lundeberg T, Lönnberg L, Svensson E.** Decrease of pregnant women's pelvic pain after acupuncture: a randomized controlled single-blind study. Acta Obstet Gynecol Scand. 2006;85(1):12-9.

11. **Malinas Y.** Les algies pelviennes au cours de la grossesse Les algies pelviennes au cours de la grossesse 1993, vol. 25, pp. 123-141

12. **Mohseni-Bandpei MA et al**. Low back pain in 1,100 Iranian pregnant women: prevalence and risk factors. The Spine Journal 2009; 9:795–801

13. **Noren L, Ostgaard S, Johansson G, Ostgaard HC.** Lumbar back and posterior pelvic pain during pregnancy: 3-year follow-up. Eur Spine J. 2002;11:267–271.

14. **Noren L, Ostgaard S, Nielsen TF, Ostgaard HC.** Reduction of sick leave for lumbar back and posterior pelvic pain in pregnancy. Spine. 1997;22:2157–2160.

15. **Olsson C, Nilsson-Wikmar L.** Health-related quality of life and physical ability among pregnant women with and without back pain in late pregnancy. Acta Obstet Gynecol Scand. 2004;83:351–357.

16. **Ostgaard HC, Roos-Hansson E, Zetherstrom G.** Regression of back and posterior pelvic pain after pregnancy. Spine 1996;21:2777-80

17. **Ostgaard HC, Zetherstrom G, Roos-Hansson E.** The posterior pelvic pain provocation test in pregnant women. Eur Spine J 1994;3:258-60.,

18. **Pennick V, Young G**. Interventions for preventing and treating pelvic and back pain in pregnancy (Review) Copyright © 2008 The Cochrane Collaboration. JohnWiley & Sons, Ltd.

19. **Robinson HS et al.** Pelvic girdle pain: associations between risk factors in early pregnancy and diasbility or pain intensity in late pregnancy: a prospective cohort study. BMC Musculoskeletal disorders. 2010, 11: 91.

20. **Robinson HS, Eskild A, Heiberg E, Eberhard-Gran M.** Pelvic girdle pain in pregnancy: the impact on function. Acta Obstet Gynecol Scand. 2006;85:160–164.

21. **Smith AC, and Cochrane S.** Does Acupuncture Have a Place as an Adjunct Treatment During Pregnancy? A Review of Randomized Controlled Trials and Systematic Reviews BIRTH 36:3 September 2009

22. **Sydsjo A, Sydsjo G, Wijma B.** Increase in sick leave rates caused by back pain among pregnant Swedish women after amelioration of social benefits. A paradox. Spine. 1998;23:1986–1990.

23. **Timsit MA.** Grossesse et douleurs rhumatologiques lombaires basses et de la ceinture pelvienne Gynécologie Obstétrique & Fertilité 32 (2004) 420–426

24. **Vermani E, Mittal R.** Weeks A Pelvic Girdle Pain and Low Back Pain in Pregnancy: A Review Pain Practice, Volume 10, Issue 1, 2010 60–71

25. **Vogler D et al.** Validation transculturelle de l'Oswestry disability index en français Annales de Réadaptation et de Médecine Physique 2008, 51(5) : 379-385

26. **Waynberger et al.** Physiologie de l'appareil locomoteur au cours de la grossesse : le syndrome douloureux pelvien de la grossesse. Revue du Rhumatisme, 2005 ; 70 : 681-5

27. **Wedenberg K,. Moen B and. Norling Å.** A prospective randomized study comparing acupuncture with physiotherapy for low-back and pelvic pain in pregnancy Acta Obstet Gynecol Scand 2000; 79: 331–335

28. **Wu WH, Meijer OG, Uegaki K, et al.** Pregnancy related pelvic girdle pain (PGP), I: terminology, clinical presentation, and prevalence. Eur Spine J. 2004;13:575–589.

XV. Liste des annexes

XV.1 Annexe 1. Score d'incapacité fonctionnelle d'Oswestry

Entourez **DANS CHAQUE PARAGRAPHE** une seule réponse, celle qui décrit au mieux votre état **au cours de la semaine passée.**

Section 1 - Intensité de la douleur

0. je ne ressens aucune douleur.
1. j'ai des douleurs très légères.
2. j'ai des douleurs modérées.
3. j'ai des douleurs assez intenses.
4. j'ai des douleurs très intenses.
5. les douleurs sont les pires que l'on puisse imaginer.

Section 2 - Soins personnels (se laver, s'habiller, etc.)

0. Je peux effectuer normalement mes soins personnels sans douleurs supplémentaires.
1. Je peux effectuer normalement mes soins personnels, mais c'est très douloureux.
2. Je dois effectuer mes soins personnels avec précaution et lenteur, et je ressens des douleurs.
3. J'ai besoin d'aide pour les soins personnels, mais j'arrive encore à effectuer la plus grande partie de ceux-ci seul(e).
4. J'ai besoin d'aide tous les jours pour la plupart de mes soins personnels.
5. Je ne peux plus m'habiller, je me lave avec difficulté et je reste au lit.

Section 3 - Soulever des charges

0. Je peux soulever des charges lourdes sans augmentation des douleurs.
1. Je peux soulever des charges lourdes, mais cela occasionne une augmentation des douleurs.
2. Les douleurs m'empêchent de soulever de lourdes charges depuis le sol, mais cela reste possible si elles sont sur un endroit approprié. (Par ex : sur une table)
3. Les douleurs m'empêchent de soulever des charges lourdes, mais je peux en soulever de légères à modérées si elles sont sur un endroit approprié.
4. Je ne peux soulever que de très légères charges.
5. Je ne peux rien soulever, ni porter du tout.

Section 4 - Marche

0. Les douleurs ne m'empêchent pas de marcher, quelle que soit la distance.
1. Les douleurs m'empêchent de marcher au-delà de 1 km.
2. Les douleurs m'empêchent de marcher au-delà de 250 m.
3. Les douleurs m'empêchent de marcher au-delà de 100 m.
4. Je ne peux marcher qu'avec une canne ou des béquilles.
5. Je reste au lit la plupart du temps et dois me traîner jusqu'aux toilettes.

Pour indication : Entrée principale de l'hôpital - Place d'Italie = 1000m

Entrée principale de l'hôpital - Entrée de la maternité = 500m

Section 5 - Position assise

0. Je peux rester assis(e) aussi longtemps que je le désire sur n'importe quel siège.
1. Je peux rester assis(e) aussi longtemps que je le désire sur mon siège favori.
2. Les douleurs m'empêchent de rester assis(e) plus d'une heure.
3. Les douleurs m'empêchent de rester assis(e) plus d'une demi-heure.
4. Les douleurs m'empêchent de rester assis(e) plus de dix minutes.
5. Les douleurs m'empêchent toute position assise.

Section 6 - Position debout

0. Je peux rester debout aussi longtemps que je le désire sans douleur supplémentaire.
1. Je peux rester debout aussi longtemps que je le désire, mais cela occasionne des douleurs supplémentaires.
2. Les douleurs m'empêchent de rester debout plus d'une heure.
3. Les douleurs m'empêchent de rester debout plus d'une demi-heure.
4. Les douleurs m'empêchent de rester debout plus de dix minutes.
5. Les douleurs m'empêchent de me tenir debout.

Section 7 - Sommeil

0. Mon sommeil n'est jamais perturbé par les douleurs.
1. Mon sommeil est parfois perturbé par les douleurs.
2. À cause douleurs, je dors moins de six heures.
3. À cause douleurs, je dors moins de quatre heures
4. À cause douleurs, je dors moins de deux heures.
5. Les douleurs m'empêchent de dormir.

Section 8 – Vie sexuelle (si présente)

0. Ma vie sexuelle est normale et n'occasionne pas de douleurs supplémentaires.
1. Ma vie sexuelle est normale, mais occasionne parfois quelques douleurs supplémentaires.
2. Ma vie sexuelle est presque normale, mais très douloureuse.
3. Ma vie sexuelle est fortement réduite à cause des douleurs.
4. Ma vie sexuelle est presque inexistante à cause des douleurs.
5. Les douleurs m'empêchent toute vie sexuelle.

Section 9 – Vie sociale

0. Ma vie sociale est normale et n'occasionne pas de douleurs supplémentaires.
1. Ma vie sociale est normale, mais elle augmente l'intensité des douleurs.
2. Les douleurs n'ont pas de répercussion significative sur ma vie sociale, excepté une limitation lors de mes activités physiques (par ex : le sport, etc.)
3. Les douleurs limitent ma vie sociale et je ne sors plus aussi souvent.
4. Les douleurs limitent ma vie sociale à mon foyer.
5. Je n'ai pas de vie sociale à cause des douleurs.

Section 10 - Voyage

0. Je peux voyager partout sans douleur.
1. Je peux voyager partout, mais cela occasionne une augmentation des douleurs.
2. Les douleurs sont bien présentes, mais je peux effectuer un trajet de plus de 2 heures.
3. Les douleurs m'empêchent tout trajet de plus d'une heure.
4. Les douleurs ne me permettent que de courts trajets nécessaires de moins de 30 minutes.
5. Les douleurs m'empêchent tout trajet, sauf pour recevoir un traitement.

Validation du score pendant la grossesse :
Robinson HS et al. Pelvic girdle pain: associations between risk factors in early pregnancy and diasbility or pain intensity in late pregnancy: a prospective cohort study. BMC Musculoskeletal disorders. 2010, 11: 91.
Mohseni-Bandpei MA et al Low back pain in 1,100 Iranian pregnant women: prevalence and risk factors. The Spine Journal 2009; 9:795–801
Gutke A et al Pelvic Girdle Pain and Lumbar Pain in Pregnancy: A Cohort Study of the Consequences in Terms of Health and Functioning Spine 2006;31:E149–E155

Validation en post partum:
Gutke A et al. Impact of postpartum lumbopelvic pain on disability, pain intensity, health-related quality of life, activity level, kinesiophobia, and depressive symptoms. Eur Spine J. EPUB 1/7/2010

Validation en Français:
Vogler D et al Validation transculturelle de l'Oswestry disability index en français Annales de Réadaptation et de Médecine Physique 2008, 51(5) : 379-385

XV.2 Annexe 2. Grille de Classification des Evénements Indésirables pour une Recherche Biomédicale portant sur un dispositif médical

Risque de la Recherche : **B** Comité de Surveillance Indépendant : Oui ☐ Non ☑

Grille d'EIG pour la Recherche Biomédicale (Art. R. 1123-54 du Code de la Santé publique)

GAME : Acupuncture pour douleurs lombaires et de la ceinture pelvienne pendant la grossesse : étude médico- économique.
Ref. P111011 - EudraCT : 2012-A00240-43

**NE PAS NOTIFIER PAR FAX au promoteur**<br>**mais à reporter sur les pages événements indésirables du CRF**<br>***(pas de remplissage du formulaire de déclaration d'EIG)***		**A NOTIFIER SANS DELAI par l'investigateur au promoteur**<br>**et à reporter sur les pages événements indésirables du CRF**<br>***(envoi du formulaire de déclaration d'EIG par fax au 01 44 84 17 99)***	
**Evénements autres**<br>EVENEMENTS POUVANT ETRE GRAVES mais non liés au dispositif médical ou aux procédures de la recherche	**Effets Indésirables Non Graves attendus**<br>Connus pour être liés :<br>au(x) dispositif médical de la recherche<br>ou aux procédures de la recherche**.**	**Effets Indésirables Graves attendus**<br>Connus pour être liés :<br>au(x) dispositif médical de la recherche<br>ou aux procédures de la recherche**.**	**Effets Indésirables Graves inattendus**
Description :<br><br>Toutes les complications de l'accouchement	Description :<br>Ce sont les incidents dus à la puncture, la littérature internationale les présente comme exceptionnels et sans gravité :<br>• Malaise vagal<br>• Rougeur<br>• Démangeaison<br>• Douleur<br>• Micro hématome	Description :<br>Ce sont les incidents dus à la puncture, mais qui ne peuvent se produire d'une part grâce à la nature du dispositif, d'autre part du fait de la localisation des points utilisés pour la recherche :<br>• Tout autre hématome (dont ceux entrainant transfusion, radiologie interventionnelle, intervention chirurgicale).<br>• Blessure d'un tronc nerveux<br>• Perforation d'organe<br>• Fracture d'aiguille nécessitant un retrait chirurgical	Cette colonne se remplira au fur et à mesure des notifications par les investigateurs, de tout évènement présentant un critère de gravité*.

***Rappel** : *Critères de gravité: **1-** Décès, **2-** Mise en jeu du pronostic vital, **3-** Nécessite ou prolonge l'hospitalisation, **4-** Séquelles durables, **5-** Anomalie ou malformation congénitale, **6-** Evénement jugé grave par l'investigateur (raison à préciser).*

Nom, prénom et signature de l'investigateur coordonnateur :	Nom, prénom et signature du responsable de l'URC :	Nom, prénom et signature du référent projet DRCD Siège :	Nom, prénom et signature du responsable pharmacovigilance :	Nom, prénom et signature du coordonnateur médical :
Dr Marc DOMMERGUES	***Pr Alain MALLET***	***Ludovic DYEN***	***Valérie ACAR***	***Pr Olivier CHASSANY***
Date :	Date :	Date :	Date :	Date :
