# Supplementary figures and images for: Cost-effectiveness of acupuncture versus standard care for pelvic and low back pain in pregnancy: A randomized controlled trial
Source: PLoS One. 2019 Apr 22;14(4):e0214195. doi: 10.1371/journal.pone.0214195 (PMC6476478; doi:10.1371/journal.pone.0214195)

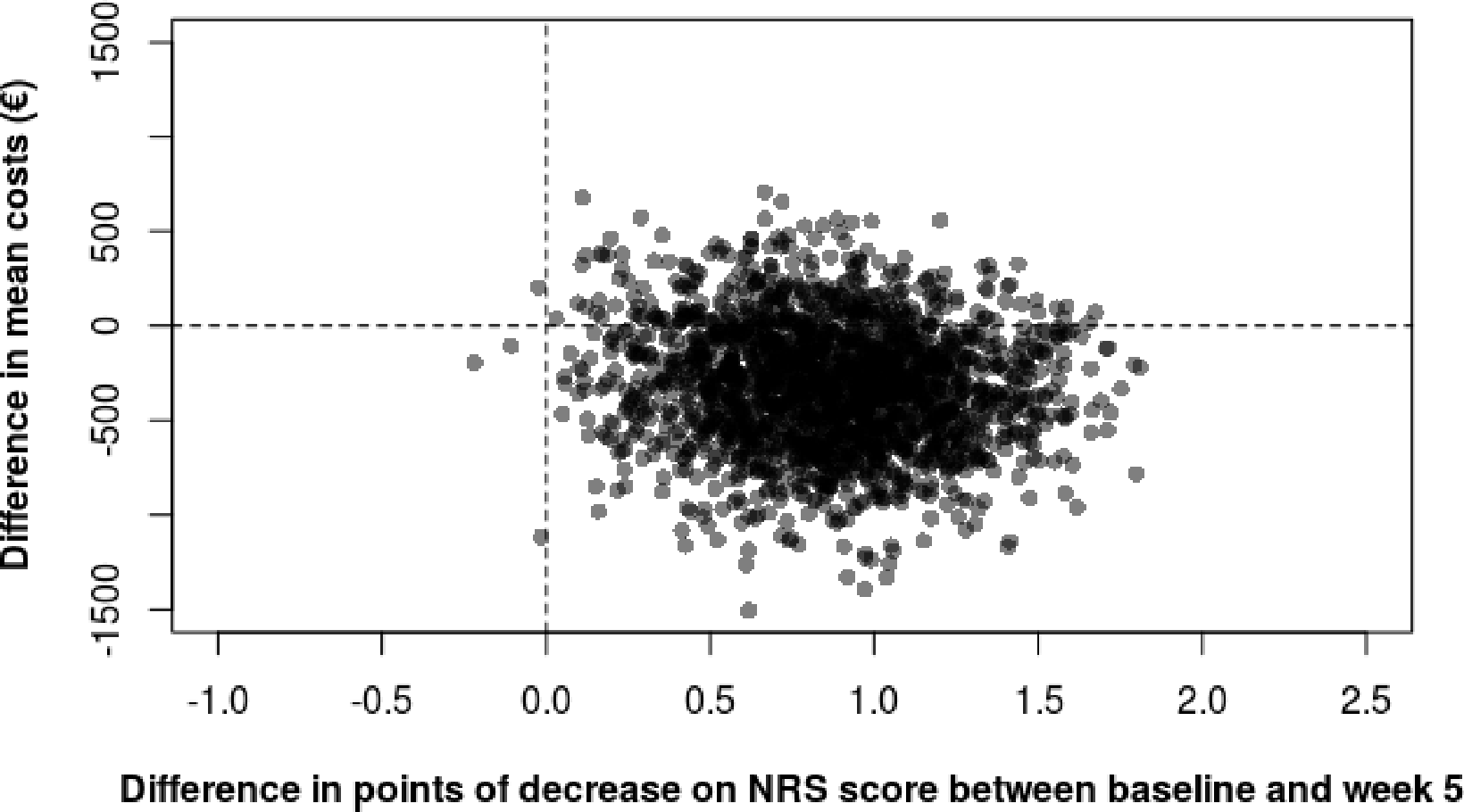

Supplement: S1 Fig — We performed 2,000 bootstrap replications of the cost effectiveness ratio. The outcome was the decrease in pain numerical rating scale between baseline and week 5 after inclusion, expressed as a difference between acupuncture and standard care. All costs were taken into account. (TIF) [file pone.0214195.s004.tif]

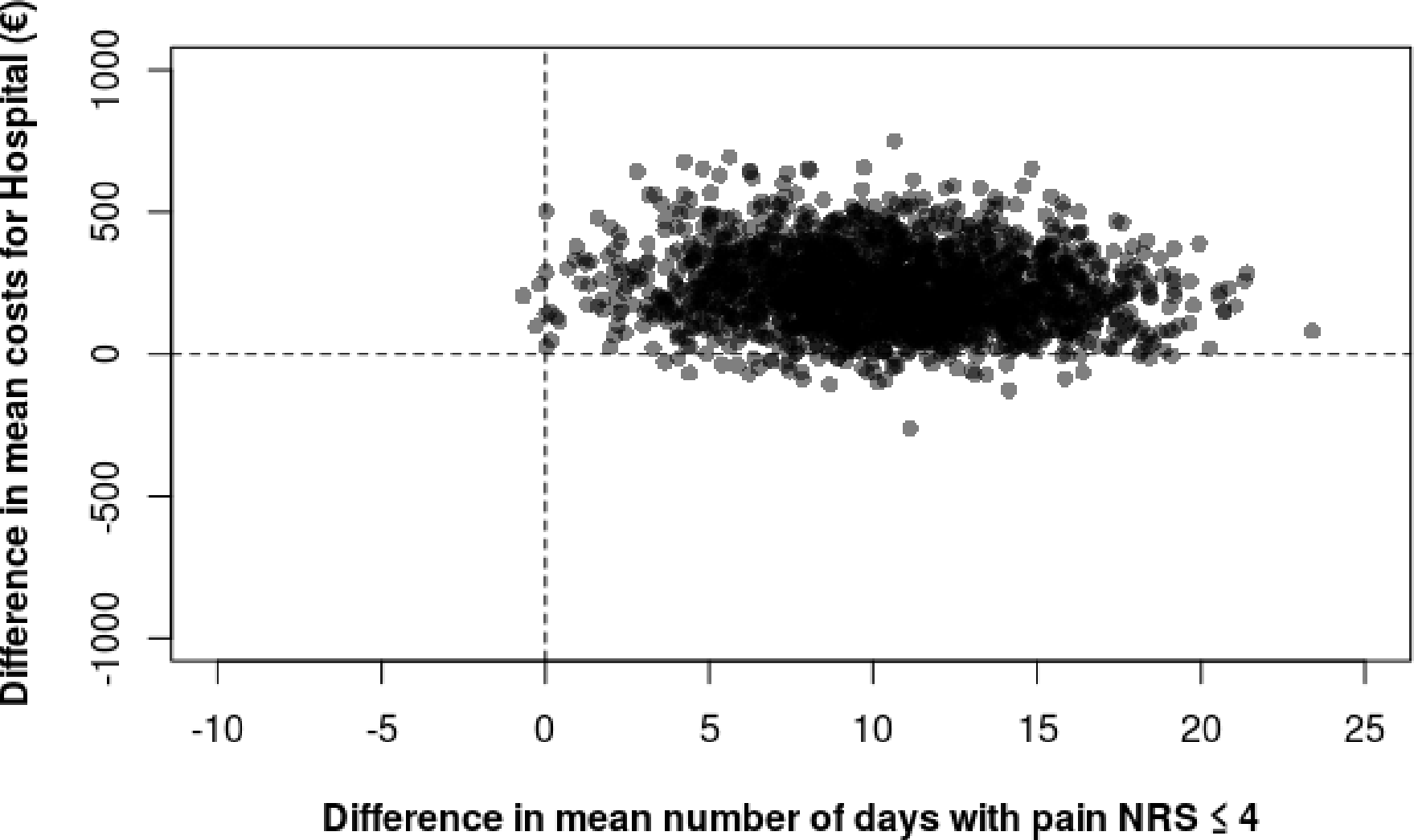

Supplement: S2 Fig — We performed 2,000 bootstrap replications of the cost effectiveness ratio. The outcome was the number of days with pain NRS ≤ 4/10 between inclusion and delivery expressed as a difference between acupuncture and standard care. Only hospital costs were taken into account. From a hospital perspective, acupuncture was more effective and more costly than routine care. (TIF) [file pone.0214195.s005.tif]

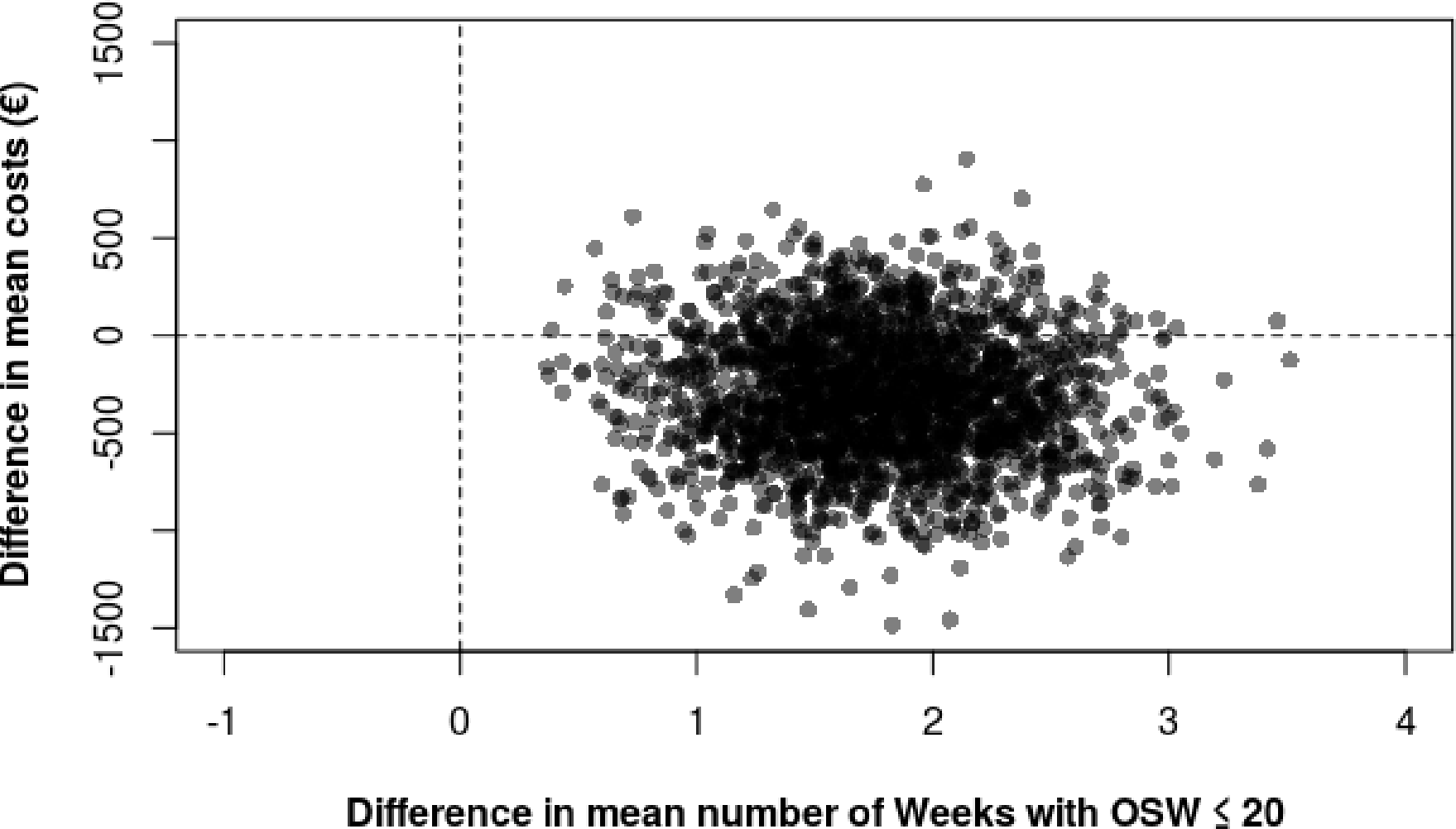

Supplement: S3 Fig — We performed 2,000 bootstrap replications of the cost effectiveness ratio. The outcome was the number of weeks with pain Oswestry score ≤ 20 between inclusion and delivery, expressed as a difference between acupuncture and standard care. All costs were taken into account. Acupuncture was more effective and less costly than standard care. (TIF) [file pone.0214195.s006.tif]
