# Supplementary material for: Cost-effectiveness of acupuncture versus standard care for pelvic and low back pain in pregnancy: A randomized controlled trial
Source: PLoS One. 2019 Apr 22;14(4):e0214195. doi: 10.1371/journal.pone.0214195 (PMC6476478; doi:10.1371/journal.pone.0214195)
Supplement: S1 Table — Traditional Chinese medicine diagnosis was based on clinical examination at inclusion. It was the first step of acupuncture treatment. (DOC) [file pone.0214195.s007.doc]

**S1 table: Traditional Chinese Medicine diagnosis criteria**

|  | **Qi kidney deficiency** | **Blood stagnation** |
| --- | --- | --- |
| Symptoms | Diffuse pain, improved by self-massage. Feeling of week knees and legs. Fatigue. Dizziness | Localized pain, increased by pressure and described as a sting.  Self-reported anxiety and stress. |
| Tongue examination | Pale and thick or red and thin | Purple or deep red coloured  Varicose veins of the tongue |
| Radial artery pulse | Pulse deficiency (radial region distal to the wrist or “ foot”, 尺). | Tense or normal pulse |
