# Supplementary material for: Cost-effectiveness of acupuncture versus standard care for pelvic and low back pain in pregnancy: A randomized controlled trial
Source: PLoS One. 2019 Apr 22;14(4):e0214195. doi: 10.1371/journal.pone.0214195 (PMC6476478; doi:10.1371/journal.pone.0214195)
Supplement: S2 Table — Baseline points were applied to all patients in the acupuncture group. Other points were used according to pain location and to traditional Chinese medicine (TCM) diagnosis. (DOC) [file pone.0214195.s008.doc]

**S2 table: Acupoints**

| **Clinical characteristics** | **Acupoints** |
| --- | --- |
| Baseline points (all patients) | 40V, *Weizhong* :  AShi points |
| Sacroiliac pain | 26V, *Guanyuanshu*:  32V, *Ci Liao*  AShi points |
| Low back pain | Inner branch of the Taiyang Bladder Channel (Shou Tai Yang 足太阳膀胱经) or Foot's Major Yang Urinary Bladder Meridian. 3 points, located bilaterally, at the level were pain is perceived, 1.5 cun distant from the spinous process. Needles inserted tangent to the skin, parallel to the meridian, directed caudally. |
| Sciatica | 30VB, *Huantiao* :  57V, *Chengshan* |
| Pain in the anterior aspect of the pelvic girdle. | 11Rn, *Hengu*  6F, *Zhongdu*  9Rn, *Zhubin* |
| TCM diagnosis of kidney deficiency | 23V, *Shenshu* :  9Rn, *Zhubin*: |
| TCM diagnosis of blood stagnation | 3F, *Tai Chong*:  Rte10, *Xue Hai* |
