# Supplementary material for: Cost-effectiveness of acupuncture versus standard care for pelvic and low back pain in pregnancy: A randomized controlled trial
Source: PLoS One. 2019 Apr 22;14(4):e0214195. doi: 10.1371/journal.pone.0214195 (PMC6476478; doi:10.1371/journal.pone.0214195)
Supplement: S3 Table — We calculated costs from the perspective of the health care system (health insurance plus hospital costs), the patient (costs at the patient’s charge), and the employer (sick leave and presenteeism). (DOC) [file pone.0214195.s009.doc]

**Table S3. Unit costs of resources in euros**

|  | Healthcare  system | Patient | Employer |
| --- | --- | --- | --- |
| **Direct healthcare costs (national estimates)**  *Acupuncture (hospital practice) per session*  *Non-urgent care visits*  *Urgent care visits*  *Physical therapist (≥45 min visit) per session*  *Acupuncture (private practice) per session*  *Hospitalization (per day)*  *Ultrasound*  *Medical transportation (return trip)* | *30*  *28*  *49*  *27*  *30*  *660*  *74*  *103* | 0  0  0  6.75  12.2  0  0  0 | -  -  -  -  -  -  -  - |
| **Direct non-healthcare costs (national estimates)**  *Relaxation per session (≥45 min)*  *Pregnancy belt*  *Osteopathy per session (≥45 min)*  *Gymnastic per session (≥45 min)*  *Massage per session (≥45 min)*  *Yoga per session (≥45 min)* | *-*  *-*  *-*  *-*  *-*  *-* | 65  40  65  15  63.5  15 | -  -  -  -  -  - |
| **Indirect non-healthcare costs (sample means)**  *Absenteeism from paid work (per day)*  *Maternity leaves (per day)*  *Presenteeism (one hour per day)* | 37  75.5  - | 28  9  - | 61  -  12.5 |
