# Supplementary material for: Cost-effectiveness of acupuncture versus standard care for pelvic and low back pain in pregnancy: A randomized controlled trial
Source: PLoS One. 2019 Apr 22;14(4):e0214195. doi: 10.1371/journal.pone.0214195 (PMC6476478; doi:10.1371/journal.pone.0214195)
Supplement: S4 Table — Social back ground was similar in the acupuncture group and in the control group. (DOC) [file pone.0214195.s010.doc]

**S4 table: Baseline characteristics, social background**

|  | | | **Acupuncture (n=96)** | **Control (n=103)** |
| --- | --- | --- | --- | --- |
| Education | *No degree*  *High school degree*  *Level 5 (2 years after finishing high school)*  *Bachelor or higher* | | 4 (4%)  19 (20%)  25 (26%)  48 (50%) | 5 (5%)  14 (13%)  33 (32%)  51 (50%) |
| Occupational category | *Executive intellectual profession*  *Employee*  *Intermediate*  *Unemployed, student* | | 19 (20%)  33 (35%)  24 (25%)  20 (21%) | 32 (31%)  31 (30%)  15 (15%)  25 (24%) |
| Carrying heavy loads | | | 28 (30%) | 30 (30%) |
| Works standing | | | 45 (48%) | 37 (37%) |
| Commuting time | | *<1h*  *1-2 hours*  *>2 hours*  *None* | 24 (25%)  35 (37%)  15 (16%)  21 (20%) | 31 (30%)  31 (30%)  16 (15%)  21 (21%) |
| Marital status | *Single*  *Married/Cohabitation* | | 6 (6%)  90 (94%) | 8 (8%)  95 (92%) |
| Geographical origin | *Caucasian*  *Sub Saharan Africa*  *Northern Africa*  *French oversee Territories*  *South America*  *Asia* | | 40 (34%)  15 (16%)  22 (23%)  10 (10%)  5 (5%)  4 (3%) | 41 (30%)  25 (24%)  21 (20%)  12 (12%)  1 (1%)  3 (3%) |
