# Supplementary material for: Cost-effectiveness of acupuncture versus standard care for pelvic and low back pain in pregnancy: A randomized controlled trial
Source: PLoS One. 2019 Apr 22;14(4):e0214195. doi: 10.1371/journal.pone.0214195 (PMC6476478; doi:10.1371/journal.pone.0214195)
Supplement: S5 Table — There was no significant difference between the acupuncture and the control group. (DOC) [file pone.0214195.s011.doc]

**S 5 table: Perinatal outcome**

|  |  | **Acupuncture (n=96)** | **Control (n=103)** |
| --- | --- | --- | --- |
| Mean gestational age at birth in weeks (SD) | | 39.6 (1.4) | 39.6 (1.3) |
| Delivery route n (%) *Vaginal spontaneous*  *Vaginal Instrumental*  *Caesarean* | | 60 (62%)  14 (15%)  22 (23%) | 65 (63%)  17 (17%)  21 (20%) |
| Onset of labour n (%) *Spontaneous*  *Cervical Ripening*  *Induction of labour*  *Planned C section* | | 67 (70%)  4 (4%)  18 (19%)  7 (7%) | 69 (67%)  4 (4%)  27 (26%)  3 (3%) |
| Premature delivery: n (%) | | 2 (2%) | 1 (1%) |
| Mean birth Weight (SD) | | 3406 (445.7) | 3327.7 (441.3) |
| Mean 5-minute Apgar score (SD) | | 9.7 (0.8) | 9.8 (0.8) |
| Neonatal resuscitation n (%) | | 3 (3%) | 2 (2%) |
| Admission to neonatal care unit n (%) | | 3 (3%) | 4 (4%) |
| Admission to neonatal intensive care n (%) | | 1 (1%) | 3 (3%) |
| Congenital birth defect n (%) | | 1 (1%) | 0 (0%) |

**Table S6. Utilisation of healthcare resources and cost for different payers**

|  |  | Acupuncture | | | |  | Control | | | |
| --- | --- | --- | --- | --- | --- | --- | --- | --- | --- | --- |
|  |  | Health system | Patients | Employers | Total |  | Health system | Patients | Employers | Total |
| Intervention  (acupuncture) |  | 160 | 0 | - | 160 |  | - | - | - | - |
| Hospitalisations |  | 233 | 0 | - | 233 |  | 167 | 0 | - | 167 |
| Out-of-hospital health care and drugs |  | 498 | 38 | - | 536 |  | 577 | 48 | - | 625 |
| Alternative  medicine |  | - | 180 | - | 180 |  | - | 203 | - | 203 |
| Sick Pay |  | 621 | 115 | 660 | 1396 |  | 707 | 170 | 853 | 1730 |
| Presenteeism |  | - | - | 130 | 130 |  | - | - | 222 | 222 |
| Total |  | 1512  (1286-1899) | 333  (267-410) | 790  (640-988) | 2635  (2269-3125) |  | 1452  (1247-1690) | 421  (338-522) | 1075  (869-1353) | 2947  (2494-3482) |

**Table S4. Nonspecific adverse** events

|  | **Acupuncture (n=96)** | **Control (n=103)** |
| --- | --- | --- |
| Number of patients with at least one adverse event | 29 (30%) | 30 (29%) |
| Number of adverse event Cholestasis  Gestational diabetes  Hypertension/ Preeclampsia  Unexplained transient fever  Urinary infection  Viral infection  Other infection  Threatened premature labour  Premature delivery (34-36 weeks)  Intrauterine growth restriction  Thrombopenia  External Causes  Other | 2 (5%)  12 (30%)  2 (5%)  1 (2.5%)  1 (2.5%)  7 (18%)  1 (2.5%)  1 (2.5%)  2 (5%)0  2 (5%)  1 (2.5%)  8 (20%)  0 | 0  11 (31%)  3 (8%)  1 (3%)  1 (3%)  4 (11%)  1 (3%)  1 (3%)  1 (3%)  5 (14%)  1 (3%)  3 (8%)  4 (11%) |
| Total number of adverse events | 40 | 36 |
| Adverse events with imputability of acupuncture | - | - |
| Adverse events by gravity *mild*  *moderate*  *severe* | 17  18  5 | 14  18  4 |
| Adverse events leading to hospitalization | 10 (25%) | 9 (25%) |

**Annexe**

**Table A. Sensibility Analysis: Impact of definition of day without pain (DWoP)**

AP => DWoP = Day with NRS ≤ 4 / 10

S1 => DWoP = Day with NRS ≤ 2 / 10

|  | **Primary Analysis** | | | | **S1** | | | |
| --- | --- | --- | --- | --- | --- | --- | --- | --- |
|  | Acup | Cont. | Diff (IC95) | p | Acup | Cont. | Diff (IC95) | p |
| **% Day Without Pain** | 61 % | 48 % | 13%  (3.6; 22.1) | 0,01 | 32 % | 22 % | 10% | 0.03 |

**Table B. Sensibility Analysis : Impact of Cost extreme values (S2) and employer cost hypothesis (S3)**

|  | **Primary Analysis** | | | **S2**  Extreme Values Withdrawal | | | **S3**  Employer Cost Hypothesis Withdrawal | | |
| --- | --- | --- | --- | --- | --- | --- | --- | --- | --- |
|  | **Acup** | **Control** | **∆** | **Acup** | **Control** | **∆** | **Acup** | **Control** | **∆** |
| **Hospital** | 393 | 167 | **226** | 310 | 167 | **143** | 393 | 167 | **226** |
| **Public insurance** | 1119 | 1286 | **-167** | 1074 | 1221 | **-147** | 1119 | 1286 | **-167** |
| **Out-of-pocket** | 333 | 421 | **-88** | 329 | 405 | **-76** | 333 | 421 | **-88** |
| **Employer** | 790 | 1075 | **-285** | 785 | 1054 | **-269** | 660 | 853 | **-193** |
| **Total** | **2635** | **2947** | **-312** | **2499** | **2847** | -**348** | **2506** | **2725** | **-219** |

S2 analysis without cost extreme values

S3 analysis without employer cost hypothesis

|  | **Before imputation** | | | **After imputation** | | |
| --- | --- | --- | --- | --- | --- | --- |
|  | **Acup** | **Control** | **∆** | **Acup** | **Control** | **∆** |
| **Hospital** | 412 | 185 | **227** | 393 | 167 | **226** |
| **Public insurance** | 1340 | 1458 | **-118** | 1119 | 1286 | **-167** |
| **Out-of-pocket** | 611 | 696 | **-85** | 333 | 421 | **-88** |
| **Employer** | 1228 | 1560 | **-332** | 790 | 1075 | **-285** |
| **Total** | **3591** | **3899** | **-308** | **2635** | **2947** | **-312** |

**Table C. Utilisation of healthcare resources and cost for different payers before and after imputation**

**Table D**. Minor side effects after acupuncture treatment

|  | **Numbers (%)** |
| --- | --- |
| Total of patients with at least one minor side effects  *Fatigue*  *Bruise*  *Headache*  *Gastro esophageal Reflux* | 33%  24 (25%)  8 (8%)  1 (1%)  1 (1%) |
