# Supplementary material for: Cost-effectiveness of acupuncture versus standard care for pelvic and low back pain in pregnancy: A randomized controlled trial
Source: PLoS One. 2019 Apr 22;14(4):e0214195. doi: 10.1371/journal.pone.0214195 (PMC6476478; doi:10.1371/journal.pone.0214195)
Supplement: S6 Table — We calculated costs from the perspective of the health care system (health insurance plus hospital costs), the patient (costs at the patient’s charge), and the employer (sick leave and presenteeism). (DOC) [file pone.0214195.s012.doc]

**S6 table: Utilisation of healthcare resources and cost for different payers**

|  |  | Acupuncture | | | |  | Control | | | |
| --- | --- | --- | --- | --- | --- | --- | --- | --- | --- | --- |
|  |  | Health system | Patients | Employers | Total |  | Health system | Patients | Employers | Total |
| Intervention  (acupuncture) |  | 160 | 0 | - | 160 |  | - | - | - | - |
| Hospitalisations |  | 233 | 0 | - | 233 |  | 167 | 0 | - | 167 |
| Out-of-hospital health care and drugs |  | 498 | 38 | - | 536 |  | 577 | 48 | - | 625 |
| Alternative  medicine |  | - | 180 | - | 180 |  | - | 203 | - | 203 |
| Sick Pay |  | 621 | 115 | 660 | 1396 |  | 707 | 170 | 853 | 1730 |
| Presenteeism |  | - | - | 130 | 130 |  | - | - | 222 | 222 |
| Total |  | 1512  (1286-1899) | 333  (267-410) | 790  (640-988) | 2635  (2269-3125) |  | 1452  (1247-1690) | 421  (338-522) | 1075  (869-1353) | 2947  (2494-3482) |
